# Evolution of Functional Diversification within Quasispecies

**DOI:** 10.1093/gbe/evu150

**Published:** 2014-06-22

**Authors:** Enrico Sandro Colizzi, Paulien Hogeweg

**Affiliations:** Theoretical Biology and Bioinformatics Group, Utrecht University, The Netherlands

**Keywords:** evolution, replicators, RNA, survival of the flattest, error threshold, interactions

## Abstract

According to quasispecies theory, high mutation rates limit the amount of information genomes can store (Eigen’s Paradox), whereas genomes with higher degrees of neutrality may be selected even at the expenses of higher replication rates (the “survival of the flattest” effect). Introducing a complex genotype to phenotype map, such as RNA folding, epitomizes such effect because of the existence of neutral networks and their exploitation by evolution, affecting both population structure and genome composition. We reexamine these classical results in the light of an RNA-based system that can evolve its own ecology. Contrary to expectations, we find that quasispecies evolving at high mutation rates are steep and characterized by one master sequence. Importantly, the analysis of the system and the characterization of the evolved quasispecies reveal the emergence of functionalities as phenotypes of nonreplicating genotypes, whose presence is crucial for the overall viability and stability of the system. In other words, the master sequence codes for the information of the entire ecosystem, whereas the decoding happens, stochastically, through mutations. We show that this solution quickly outcompetes strategies based on genomes with a high degree of neutrality. In conclusion, individually coded but ecosystem-based diversity evolves and persists indefinitely close to the Information Threshold.

## Introduction

In the classical formulation of quasispecies theory, populations are modeled as collections of mutationally interconnected genotypes with different growth rates. The outcome of the mutation–selection dynamics is a stable distribution of closely related genotypes: A quasispecies.

The structure of the quasispecies depends on the particular choice of the mutational scheme and the relative growth rates of the mutants, features often summarized together in the concept of fitness landscape. In a “steep” landscape the overall growth rate drops drastically in the close mutational neighborhood, whereas in a “flat” one many genotypes have similar fitness. As selection targets the population with the highest average growth rate (Schuster and Swetina 1988), in a fitness landscape with both steep and flat regions, the quasispecies can be distributed in the flatter parts at high mutation rates, even if the fittest genotypes there have lower growth rates. This effect is called “survival of the flattest.”

In these models, genotypes are defined solely in terms of growth rate, and fitness landscapes are static and predetermined. Alternatively, replicators can be characterized by an explicit genotype, and a genotype-to-phenotype map. To this end, a biologically grounded instance consists of “coding” genotypes as RNA-like sequences, and phenotypes by RNA folding to secondary structure, thus colocating information and functionality on a single molecule. RNA folding as a genotype-to-phenotype map is known to 1) be very rugged (Fontana et al. 1993; Huynen et al. 1993) and 2) have intertwined neutral networks which percolate throughout the entire genotype space (Schuster et al. 1994; Huynen 1996). A population of replicators that evolves on a neutral network eventually spends most of the time on its highly connected regions, provided that mutation rate and population size are large enough (Van Nimwegen et al. 1999). Hence, neutrality increases automatically in the long-term evolution of replicators with explicitly defined genotypes and phenotypes.

A quasispecies can be maintained in the system only if mutation rate is below a threshold value, the Error Threshold, above which the effect of too frequently arising mutants cannot be counteracted by selection, Darwinian optimization breaks down, and the quasispecies delocalizes. A consequence of the Error Threshold is that, if the per-base mutation rate is constant, longer sequences suffer from mutations more than shorter ones, and there exists a maximum length a sequence can sustain above which the accumulation of deleterious mutations cannot be prevented: The Information Threshold (Eigen 1971).

The Information Threshold poses a serious limit on the evolutionary accumulation of information in a genome. Earlier approaches to overcome such limitation consisted of modeling different types of replicators interacting with each others, which, collectively, would integrate more information than each individual species (e.g., the hypercycle; Eigen and Schuster 1978). By introducing interacting replicators, the problem of information integration is taken from the quasispecies level to the ecosystem level. However, in well-mixed conditions, these systems are evolutionarily unstable to species that benefit from being replicated without giving replication, that is, parasites. Not so if some form of compartmentalization is taken into account, be it explicit (as in Szathmáry and Demeter 1987), or emergent as a consequence of the dynamics in discrete spatially extended systems, for example, in the form of spiral waves (Boerlijst and Hogeweg 1991), or travelling waves of replicators/parasites (Hogeweg and Takeuchi 2003). More generally, spatial pattern formation has been shown to have important consequences for the eco-evolutionary dynamics of a system (e.g., Takeuchi and Hogeweg 2009).

Eigen and Schuster (1978) (part B) considered functionally cooperating partners belonging to different lineages to be the only possible solution to the problem of integrating more information in a system (i.e., the hypercycle). Quasispecies-based solutions were excluded on the rationale that genotypic kinship relations cannot confer functional phenotypic coupling.

Here, in stark contrast, we show that a single quasispecies can integrate a large amount of information at high mutation rates, and that it behaves functionally like an ecosystem. In particular, we extend the analysis of a recently developed model (Takeuchi and Hogeweg 2008) by focusing on quasispecies dynamics and the survival mechanisms of interacting replicators: We characterize the evolved quasispecies not only in terms of replication rates but also by the emergent functional roles of mutants.

The Results section is structured as follows: 1) A quasispecies which survives at high mutation rates is evolved; 2) the master sequence is determined and it is shown that replication rate is neither maximized, nor can it alone explain the survival of such sequence; 3) it is established that, counter to expectations, the evolved quasispecies is exceptionally steep, that is, neutrality is low and most mutants are not viable; 4) we propose a functional classification of nonviable phenotypes and the evolved quasispecies is explored in depth; and 5) the role of the functional classes is analyzed in simplified models as well as by their spatial distribution. Finally, results are put together and the unified picture of a “functional ecosystem,” populated mostly by nonviable mutants, is presented. As results are presented by means of one case study, the generality of the results and the (rare) qualitatively different outcomes close the Results section.

## Materials and Methods

### Model

**Fig. 1.—:**
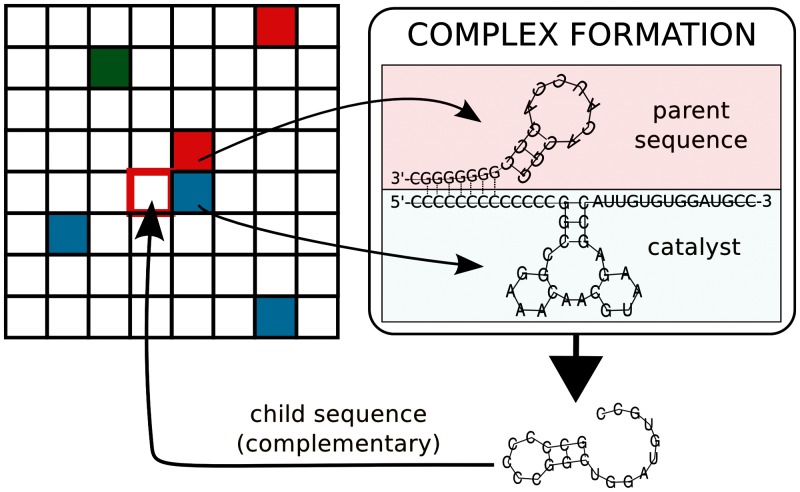
Schematic representation of complex formation and replication: Two adjacent sequences form a complex based on sequence similarity; if the molecule binding with the 5′-dangling end is catalytic, in presence of empty space, replication takes place and the complementary of the other sequence is produced. Mutations may happen at this step. In the model, all sequences are 50 nt long, here they are depicted shorter for clarity.

The system is a spatially extended, individual oriented, Monte Carlo simulation model. Individuals consist of RNA-like strings of constant length (50 nucleotides) which are folded (to minimum free energy secondary structure, with Vienna Package version 1.7; Hofacker et al. 1994). They are located on a two-dimensional square grid of 512 × 512 cells with toroidal boundaries (based on CASH libraries; de Boer and Staritsky 2000). Each cell of the grid can be occupied by at most one individual. Figure 1 gives a representation of the model. One Monte Carlo step is as follows: All cells are chosen in random order and, if not empty, 1) complex formation or complex dissociation may happen; 2) if in complex with a catalytically active molecule (see below), and in presence of empty space, a sequence can be replicated and the complementary sequence is generated (after which the complex breaks apart), mutations may occur; 3) diffusion takes place as one step of random walk; and 4) sequences can decay, that is, the cell they occupy turns to empty. Suppose *X* is a catalytically active molecule and *Y* is not, then, schematically:

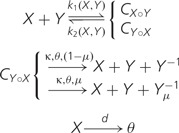

where *C* is a complex molecule, *θ* represents empty space (which constitutes the resource for replication), $Y^{-1}$ is the complementary sequence (the subscript *μ* refers to the mutated sequence), *κ* is the replication rate, and *d* is the decay rate. $k_{1}(X,Y)$ and $k_{2}(X,Y)$ are the probabilities that, respectively, complex formation and complex dissociation happen. Complex formation happens by binding the 5′-dangling end and the 3′-dangling end of two molecules adjacent on the grid. The probability of binding depends on the complementarity of the two dangling ends: The two stretches are aligned by sliding one strand on the other, to find the minimum energy score ($G_{\text{min}}(X{}^{\circ}Y)$), calculated as the minimum sum of the contributions of base pair matches in a continuous stretch (i.e., gaps are not allowed). *G*–*C* contribution is −0.15, *A*–*U* is −0.1 and *G*–*U* is −0.05, the contribution of all other base pairs is zero. The probability of binding is then $P_{X{}^{\circ}Y}=1-\exp(G_{\text{min}}(X{}^{\circ}Y))$. As two molecules can form complexes in two ways (because either can be at the 5′-end), the probability of binding is calculated for both configurations (so, in the same way: $P_{Y{}^{\circ}X}=1-\exp(G_{\text{min}}(Y{}^{\circ}X))$). If $P_{X{}^{\circ}Y}+P_{Y{}^{\circ}X}>1$ the two probabilities are normalized, that is, $k_{1}(X{}^{\circ}Y)=P_{X{}^{\circ}Y}/(P_{X{}^{\circ}Y}+P_{Y{}^{\circ}X})$, otherwise $k_{1}(X,Y)=P_{X{}^{\circ}Y}$. The probability of complex dissociation is $k_{2}(X,Y)=1-k_{1}(X,Y)$.

Quantitatively, if two dangling ends match only for a *G*–*C* pair, the probability of complex formation is negligible (≈0.015), whereas complex dissociation is very likely (1−0.015=0.985); moreover, it takes at least five *G*–*C* pairs (or seven *A*–*U* pairs) for the probability of complex formation to be larger than that of complex dissociation ($P_{X{}^{\circ}Y}>0.5\Leftrightarrow G_{\text{min}}(X{}^{\circ}Y)<-0.694$, which, for *G*–*C* only pairs is true when G(5×(C°G))=−0.75, and for *A*–*U* pairs when G(7×(A°U))=−0.7).

Catalytic activity consists of being able to replicate other molecules. A sequence is defined as catalytic if it folds into a predetermined, coarse grained secondary structure, arbitrarily chosen to consist of a multiloop which connects a stem to two hairpins, ((((H)S)((H)S)M)S), in Shapiro notation (as implemented in the Vienna Package). Two more conditions need to be satisfied for replication to take place: The catalytic molecule must be engaged in complex with its 5′-dangling end and empty cell must be present in the neighborhood of the complex. The sequence complementary to that at the 3′-end of the complex is generated, folded, and located on the empty cell. The only mutations implemented are substitutions, which happen with a per base probability *μ*. The replication rate, *κ* is set to 1 (i.e., replication depends only on complex formation and the availability of empty space), the decay probability *d* is set to 0.03. A small probability of not moving (*P* = 0.1) is introduced for complex molecules, to take into account their slower diffusion.

### Phenotype Recognition and Classification

For the analysis of the simulation output we have developed a classification of individual molecules which is based on presence/absence of catalytic domain, as well as on 5′- and 3′-dangling ends. Considering both a sequence and its complement, which together define a genotype, any phenotype can be coarse-grained to 6 bits of information, meaning that there exist $2^{6}=64$ possibilities. If a dangling end is short, a molecule would have a small probability of forming a complex. In order to classify a molecule as having/not having a dangling end, the complementary stretch is generated, and the probability of complex formation is calculated as above. We set a threshold for the energy score of the complex: If G≤−0.75 (corresponding to a probability ≈0.5) then the molecule is classified as having that dangling end, otherwise, as not having it.

**Fig. 2.—:**
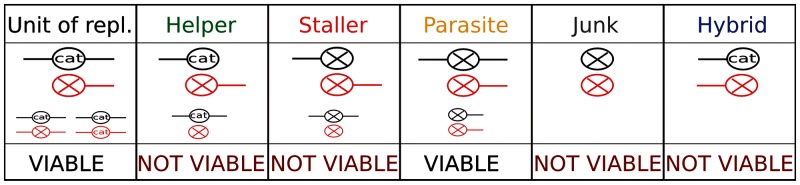
Classification of the mutational classes. Phenotypes are determined for pairs of complementary sequences (black: + strand, red: − strand), and are coarse grained to presence/absence of 5′-dangling end, catalytic structure, 3′-dangling end. As different phenotypes may fall in the same functional class, some of all the possibilities are depicted (the larger ones are the most frequent configurations). “Helpers” are defined by having a 5′-dangling end and the catalytic structure on at least one (and the same) strand, and not having a 3′-dangling end in both strands. “Stallers” are defined by having a 5′-dangling end but no catalytic structure on at least one (and the same) strand, and not having a 3′-dangling end in both strands. “Junk” are sequences that do not have any 5′-dangling end on either strands, and no 3′-dangling end on at least one. “Hybrid” sequences display a helper-like phenotype on one strand and staller-like on the other.

Phenotypes with minor differences can be grouped into phenotype classes, which can be further joined by integrating functional considerations. In figure 2, the proposed classification is presented. The dependence on a particular threshold value to recognize a dangling end as such (G≤−0.75) is minimal in the evolved system (within a reasonable range; see supplementary fig. S1, left pane, Supplementary Material online). In contrast, the distribution of the functional classes in random sequences does depend on it (right pane).

A necessary (intrinsic) condition for genotypes to be replicated is to have a 3′-dangling end on both strands. However, in order to give replication, a sequence must fold into the catalytic structure and have a 5′-dangling end. A “unit of replication,” is a viable pair of complementary sequences, of which at least one is able to give replication. It is trivial to observe that, for a minimal system to persist, units of replication have to be present. Nonetheless, the minimum viable phenotype consists of a pair of sequences that do not fold into the catalytic structure, have 3′-dangling end, but no 5′ ones. Such phenotype could exploit the units of replication for catalysis, and as such would be a parasite.

Units of replication and parasites are the only two viable classes. Among the nonviable ones, we define “helpers” as those sequences which can replicate other molecules but cannot be replicated, “stallers” can engage molecules in complex, but can neither replicate them nor be replicated, “junk” cannot form complexes (these phenotypes are mostly inert), and “hybrids” sequences display a helper-like phenotype on one strand and staller-like on the other.

## Results

### Evolving Persistence to High Mutation Rates

Sequences have to be evolved in order to withstand high mutation rates because initializing the system already at high mutation rates (μ≥0.013) with randomly generated units of replication leads to a quick extinction (see below). Starting from low mutation rates, each time a sequence is mutated, a small positive random number is added to its mutation rate (if the distribution is unbiased and negative numbers can be drawn, mutation rate decreases). During the evolutionary run, the system displays various dynamic regimes which can be characterized by the structure and the stability of the evolving ecosystem (see supplementary material and fig. S2, Supplementary Material online). The dependence of the number of species and ecosystem structure on mutation rate is in line with Takeuchi and Hogeweg (2008): Although for lower mutation rates multiple lineages coexist in the field, when mutation rate is sufficiently high (μ≥0.014) only one quasispecies is present. At this point, the simulation is continued by setting the value of *μ* to constant. For the case we focus on, μ=0.015, which corresponds to a probability of at least one mutation happening per replication event $1-{\left(1-\mu \right)}^{\nu }=0.53$ (with *ν* [length of a sequence] =50 nt).

### The Master Sequence of the Quasispecies

**Fig. 3.—:**
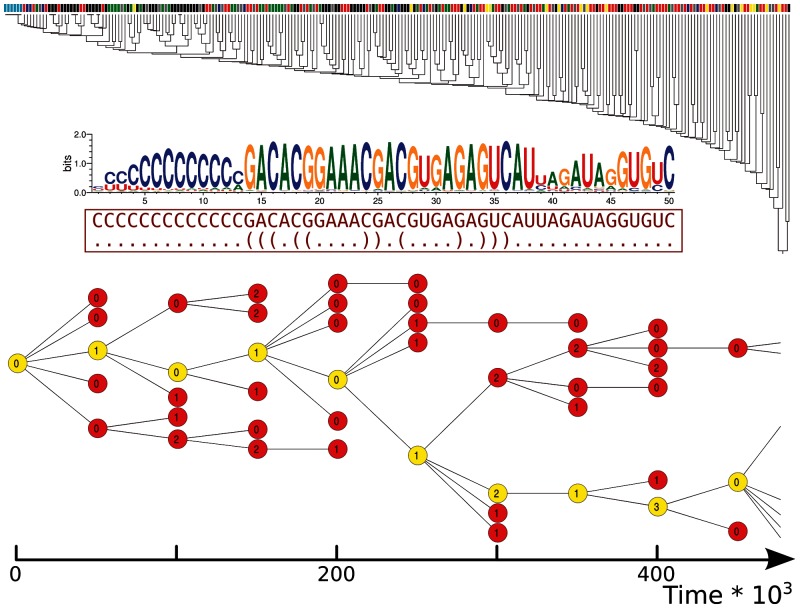
Evolved quasispecies: Clustering, sequence logo, consensus sequence, and ancestor tree. Top: Clustering, sequence logo, and most abundant sequence with its secondary structure (in dots-and-brackets notation) of the quasispecies at μ=0.015. In total, 1,000 sequences are randomly sampled from the population at one time point, clustered (500 are displayed for clarity) and the sequence logo is generated (sampling at different time points, or larger samples do not produce qualitatively different results. Colors on the leaves correspond to the functional class in which sequences fold (see Materials and Methods). Cyan: most abundant sequence, black: units of replication, green: helpers, red: stallers, grey: junk, blue: hybrids. The tree is visualized with iTOL (Letunic and Bork 2011). Bottom: Ancestors tree over the first $450\times 10^{3}$ time steps of a simulation run (the simulation is initialized with a homogenous population of master sequences, it consisted of $\approx 2.5\times 10^{6}$ time steps, after which it was interrupted). The rest of the simulation as well as different runs shows qualitatively the same pattern. The tree is built so that the nodes at a given time step are every individual’s ancestor $50\times 10^{3}$ time steps later, edges connect lines of descent; yellow nodes are those in the line of descent that persists until the end of the simulation, in red the others. Numbers mark the Hamming Distance of the ancestor from the master sequence. The tree is visualized in Cytoscape (Smoot et al. 2011).

Clustering the sequences reveals that only one quasispecies is present in the field, with a high degree of sequence similarity (fig. 3, top). The consensus sequence is also the most abundant genotype occurring, thus the master sequence, and the center of the quasispecies. The inspection of the ancestors tree (fig. 3, bottom) confirms it, as such sequence is the most frequently observed ancestor of every other molecule (cf. Hermisson et al. 2002). However, at any time point, several sublineages coexist and compete even for long time periods before going extinct. Along the line of descent (in yellow), as well as in the other lineages (in red), the presence of the master sequence is intermittent, and the ancestor of the lineage becomes a close mutant. Nonetheless, back mutations restore the original sequence in the long run. This is an indication that the system is close to the Error Threshold (cf. metastable states close to the Error Threshold in Takeuchi and Hogeweg 2007a).

### Replication Rate

As in quasispecies theory fitness is defined (solely) by replication rate, we calculate the replication rate of the master sequence and analyze to which extent it is maximized in the evolved quasispecies.

The phenotype associated with the master sequence is catalytic and has two dangling ends. The 5′ one is composed exclusively of C’s, whereas the 3′-end lacks a clear pattern in sequence composition. The complementary strand is not catalytic, with a closed 5′-end, and a 3′-dangling end with only G’s. Taking into account both strands, the overall phenotype class is that of a unit of replication. In order to replicate, the catalytic strand has to be able to form a complex with both itself and the complementary sequence. Clearly, the 5′-end of the catalytic strand matches perfectly with the 3′-end of the opposite strand (the probability of complex formation is ≈0.86). However, forming a complex with another catalytic strand poses a problem: Having too many G’s on the 3′-end, which would, in principle, ensure a high complex formation probability, could cause a molecule to fold on itself (forming a stem), thus leaving no dangling ends. This explains the intricate mixture of nucleotides at the 3′-dangling end of the catalytic strand. The probability that two catalytic strands of the master sequence form a complex is 0.90.

**Fig. 4.—:**
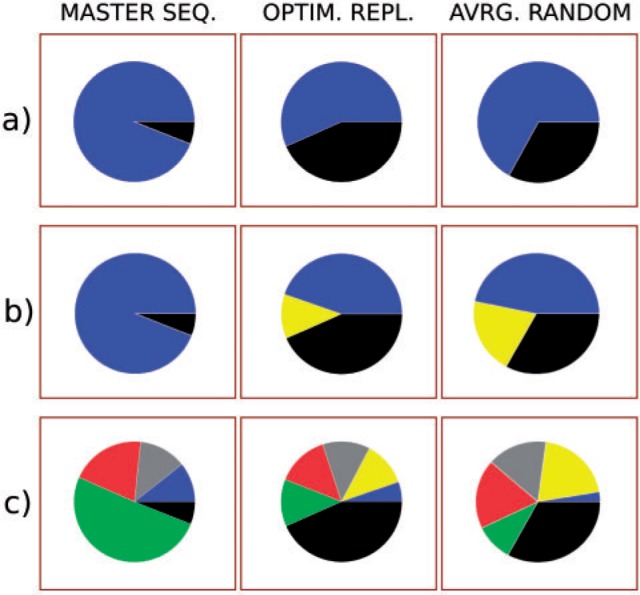
Hamming Distance = 1 mutational neighborhood of the evolved master sequence (left), one typical maximally fit sequence after evolutionary optimization for replication rate (centre), the average random unit of replication (right). The piecharts show the fraction of mutants that fold into the various functional classes. (*a*) Units of replication (in black), that is, neutrality versus the other foldings (in blue). (*b*) As above, with, in yellow, the fraction of mutants that fold into parasite. (*c*) The full mutational neighborhood. Colors of functional classes are the same as for the cluster tree. The sequence of the optimized unit of replication is AAAACGUGUAAAGGAGCGAAUCGCAGGCAGAGCCACCAUAAAAGUUAUUA. Random units of replication are obtained by generating 10^6^ random sequences and screening their function: 228 units of replication are found. All HD = 1 mutants of the latter are generated and the fractions of mutants folding into each functional class are determined.

The self-replication rate of the master sequence is rather high, due to its long dangling ends and the *C*–*G*-based strategy for complex formation. To test whether self-replication is maximized we implemented an evolutionary optimization algorithm that selects sequences for replication rate, without considering any interaction between genetically different individuals. Catalytically perfect units of replication evolve quickly by using an evolutionary optimization algorithm that selects sequences for replication rate. Genotypes consist of a pairs of complementary sequences, both catalytic, for which the total probability of binding both the same strand and the complementary is equal to 1 (i.e., when μ=0, the only limit to growth is diffusion; see Materials and Methods). Sequence composition on the dangling ends of units of replication evolved with this method is far from being dominated by C’s, rather, A’s and U’s (interspersed within each tail) are the most frequently observed nucleotides (see caption of fig. 4).

As the optimized sequences achieve the highest absolute degree of optimization for replication rate, whereas those in the full model do not, the former does replicate faster also in the full model, when μ=0. However, all the sequences tested quickly go extinct for μ≥0.013, where the master sequence easily survives.

A unit of replication which has both strands catalytic is never observed in the model, possibly because both its strands can be exploited by parasites as well as other (non symmetric) units of replication. Moreover, from a pool of randomly generated units of replication, we found that those catalytic on both strands are the minority (37%), and for only 4% of the sequences each strand replicates the other better than self. Units of replication optimized for replication rate so that only one strand can be catalytic achieve slightly higher replication rates than those in the full model. Moreover, they are not biased in nucleotide composition and they invariably go extinct at high mutation rates (data not shown).

In conclusion, the selection of the master sequence cannot be explained by considering exclusively its (partially optimized) replication rate, or the structure of its dangling ends.

### The Mutational Neighborhood of the Master Sequence

Having excluded that optimization at the level of replication rate is the sole outcome of evolution, we turn to study the effect of interactions within the quasispecies, that is, the interactions with the mutants of the master sequence. We first consider the viable mutants, that is, neutral ones and parasites, then we characterize the rest of the mutational neighborhood.

#### Neutrality

**Fig. 5.—:**
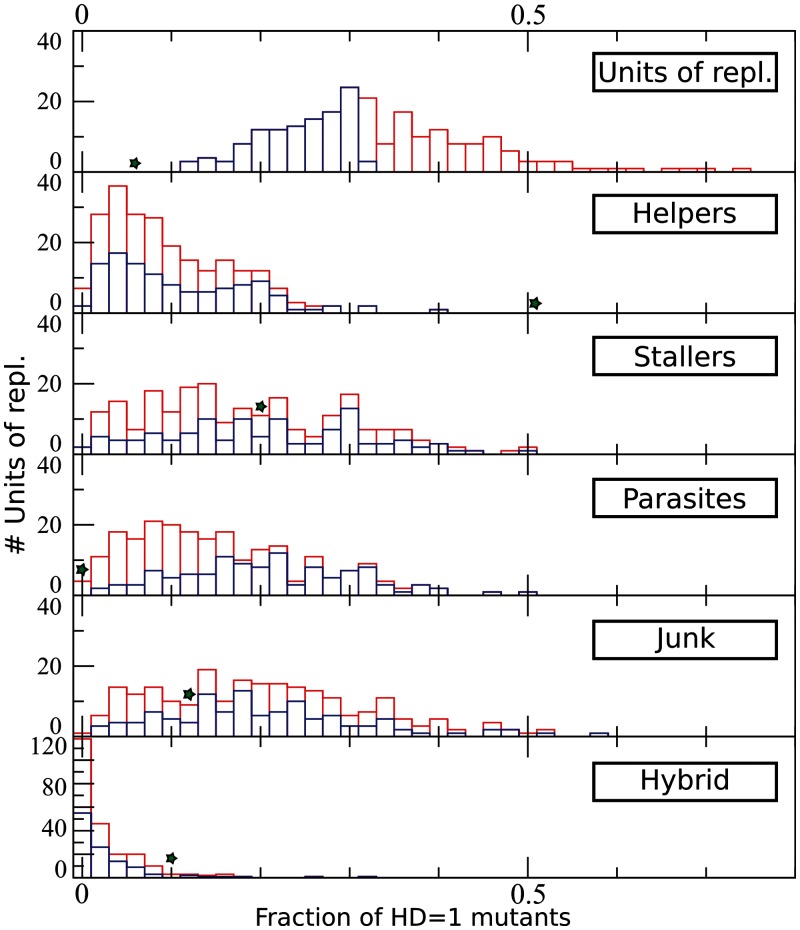
Distribution of HD = 1 mutants for random sequences and for the master sequence. Each histogram displays the distribution of number of units of replications for their fraction of mutants that fold into the functional class indicated (bin size = 0.02). In blue, the distribution of the 50% least neutral sequences among different classes; in red, the 50% most neutral. Green stars: fraction of mutants of the master sequence belonging to the class indicated.

The fraction of all the Hamming Distance (HD) = 1 mutants of the master sequence that fold into units of replication is $\lambda_{\text{ms}}=0.06$ (note that a broad definition of neutrality is adopted, as folding into a unit of replication suffices, and replication rate is not taken into account). In comparison, the average degree of neutrality for random units of replication is $\lambda_{\text{r}}=0.33$ and for a typical unit of replication obtained by optimizing replication rate is even larger ($\lambda_{\text{opt}}>0.40$, fig. 4, top row). Interestingly, the master sequence seems to belong to a “steep” (as opposed to “flat”) quasispecies. The master sequence is also nonmodular: The fraction of units of replication obtained when mutating only the dangling ends of the catalytic strand is only slightly higher than the neutrality of the whole sequence ($\lambda_{\text{tails}}=0.11$). Moreover, the master sequence is a far outlier with respect to the neutrality distribution for random units of replication (fig. 5, first pane).

#### Parasites

In this model, parasitic lineages readily evolve at lower mutation rates and, because of spatial structuring, they do not destroy the ecosystem (supplementary material, Supplementary Material online; Takeuchi and Hogeweg 2008). Strikingly, the chances of generating a parasite as a HD = 1 mutant of the master sequence are exactly zero (0.0005 at HD = 2), despite the fact that a single mutation disrupting the catalytic structure of a unit of replication could suffice. In contrast, the fraction of parasites in the HD = 1 mutational neighborhood of both random units of replication and the ones optimized for replication rate is much higher (fig. 4, center row). When these sequences are inoculated in the full model at high mutation rates, parasites are going to be generated often and, being very similar to the unit of replication they originate from (HD = 1), they become strong competitors for getting replicated. As close mutants of units of replication, they are not spatially separated (as it happens in, e.g., travelling waves) and lead the system to extinction, similarly to the situation in a well-mixed system (cf. e.g., Takeuchi and Hogeweg 2007b). This explains why these sequences fail to survive despite having a high replication rate. In conclusion, long-term evolution at high mutation rates minimizes the chances of producing parasites in the quasispecies.

So far, only 6% of the HD = 1 mutational neighborhood has been characterized, considering the low degree of neutrality of the master sequence ($\lambda_{\text{ms}}=0.06$) and the fraction of its mutants that turn into parasites ($\lambda_{P}=0$). This completes the description of the viable mutants. In the next section, the remaining 94% of nonviable mutants are better characterized.

#### The HD=1 Mutational Neighborhood

In the Materials and Methods section, the classification of all the possible phenotypes has been introduced. Here we analyze the distribution of such classes in the mutational neighborhood of the master sequence, to conclude that the evolved quasispecies is selected for the peculiar distribution of its mutants.

In figure 4, bottom row, the relative fractions of functional classes arising as mutants of the master sequence are compared with those of optimized sequences and random ones (as before). Helpers make up to about 50% of the mutational neighborhood of the master sequence, in contrast to the case of random sequences (≈10%) and those optimized for replication. The master sequence does not seem to differ much from random sequences for what regards junk, stallers, and hybrids.

In figure 5, the mutants of the master sequence are compared with the full distribution of mutants from random units of replication, rather than just with the average values. The green star in each subplot represents the frequency of each functional class in the HD = 1 mutational neighborhood of the master sequence. The degree of neutrality of the master sequence ($\lambda_{\text{ms}}=0.06$), the fraction of its mutants that fold into parasites ($\lambda_{P}=0$) or helpers ($\lambda_{H}=0.51$) are far outliers of their respective “null” distributions: The first two are underrepresented, whereas helpers are overrepresented. Stallers, junk, and hybrid sequences in the neighborhood of the master sequence seem not to be significantly different from random.

It could be the case that, as the total number of possible mutants produced is constant (i.e., three possible substitutions*sequence length = 150 mutants), the lower degree of neutrality in the master sequence would allow for more different kinds of functional classes as a side effect. To address this caveat, the population of random units of replication is split into two groups: The 50% least neutral sequences (blue in fig. 5) are separated from the 50% most neutral (red), and the two groups are compared for the other functional classes. We find no evidence that such is the case, as the two groups distribute roughly in the same way for all functional classes. Altogether, this indicates that the properties of the HD = 1 mutational neighborhood of the master sequence are evolved and that the master sequence is selected for its mutational neighborhood which minimizes neutrality and frequency of parasites, as well as maximizes the amount of helpers.

Comparing the mutational neighborhood of random units of replication with the global occurrence of functions in genotype space (supplementary material and fig. S1, right pane, Supplementary Material online), we observe that the global occurrence of helpers and hybrids is limited, which explains why they are so infrequent as mutants of random units of replication. Instead, it is remarkable that some units of replication have only few stallers, junk, or parasites, given their abundance in the genotype space. However, as mentioned above, most of the sequences in these functional classes might be unreachable with few mutational steps from a unit of replication. From which we conclude that the genotype space from the “mutational point of view” of a particular phenotype looks biased from the global picture. Of course, selection can act on the former, and not on the latter.

#### The Mutational Neighborhood at Larger Distance

Units of replication arise as mutants of the evolved master sequence, and, in turn, make more mutants. Analyzing what the mutational neighborhood is like at higher Hamming Distances allows us to understand to which extent of the mutational neighborhood selection pressure reaches.

**Fig. 6.—:**
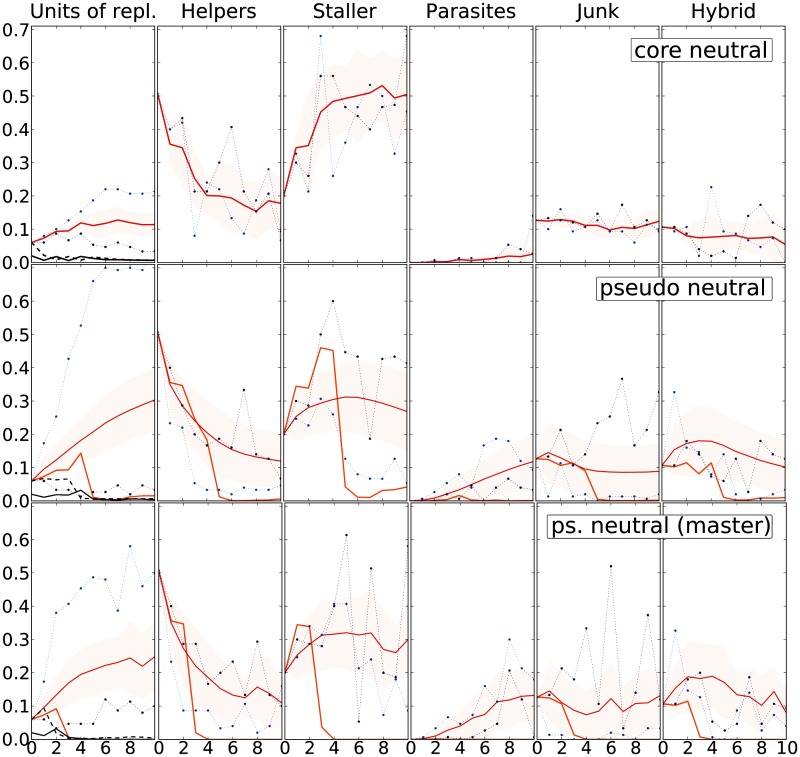
Mutational neighborhood of the master sequence at higher Hamming Distances. First row: Fully neutral mutants (units of repl. with replication rate as high or higher than that of the master sequence). Second row: Pseudoneutral mutants (units of repl. with repl. rate lower than that of the master sequence). Random sampling for units of replication if more than 10^4^ are found in both cases. Third row: Pseudoneutral mutants sampled from the master sequence at Hamming Distances from 1 to 10 (if the total number of units found exceeds 10^4^ or if, after 10^5^ tries, no more units are found, the algorithm moves on to the next Hamming Distance value). In the first row: Continuous red line: average fraction of phenotypes in each class. In the second and third row: Thick red line: average fraction of phenotypes in each class for pseudoneutral mutants; orange line: average fraction of mutants for core neutral units of replication. For all rows: Shaded area: between +/− standard deviation. Continuous black line: fraction of total mutants that are core mutants. Dashed black line: fraction of total mutants that are pseudoneutral mutants. Blue dotted line: mutational neighborhood of the sequence with lowest neutrality at a certain Hamming Distance. Light blue dotted line: as before, except for highest neutrality.

The first search to higher Hamming Distances is performed by selecting units of replication that have a replication rate as high as (or higher than) that of the master sequence. Starting from the master sequence, the average mutational neighborhood of these units of replication, at progressively higher Hamming Distances, is plotted in figure 6 (core neutral mutants). The frequency of units of replication mutants of neutral sequences increases slightly and seems to saturate for HD > 4, whereas the fraction of core neutral mutants among them remains extremely low. The fraction of helpers drops quickly, whereas, symmetrically, the fraction of stallers increases. Very few parasites are present, and there seems to be little variation for junk and hybrid sequences. Taking into account that mutants in the close mutational neighborhood are also close in space in the full system (because replication is a local process), the picture that emerges is that the master sequence is able to outcompete neutral mutants by being replicated more often than anyone else by helpers, while being hindered less than every other unit of replication by stallers.

A second search is performed by selecting any unit of replication (pseudo neutral in fig. 6, second row). The main differences with the results above are the marked increase in units of replication at higher Hamming Distances and the larger amount of parasites, whereas stallers do not increase as much as in the previous case. Although units of replication are increasingly neutral at higher Hamming Distances, they do not outcompete the master sequence.

Finally, a third procedure is implemented by sampling the mutants of the master sequence at progressively higher Hamming Distances. This procedure is implemented for the sake of completeness: Some units of replication may be generated only with multiple mutations (hence, they would not be taken into account by the previous procedures) and may contribute to the overall shape of the landscape. However, the mutational neighborhood emerging from this method is very similar to the one described above (fig. 6, third row).

Altogether, the analysis of the mutational neighborhood suggests that selection acts to shape and finely tune the mutational neighborhood in such a way that small genotypic variations (single substitutions) have large effects on the functional role of the phenotypes. Moreover, interactions with strong competitors are minimized, as they are mediated by either noncompetitive units of replication (which dilute the latter) or nonviable sequences. The distribution of the nonviable mutants of the master sequence is shaped in such a way to contribute to the replication of the master sequence itself (the helpers at low Hamming Distance) or hinder competitors (stallers at high Hamming Distance). The mutational neighborhood of units of replication selected with the second method becomes more and more similar to that of random units of replication the higher the Hamming Distance. Above, we have seen that random units of replication are not viable at high mutation rates; hence, they cannot be competitors of the master sequence. The lack of a structured mutational neighborhood (especially for the abundance of parasites) explains their quick extinction (see above).

### Spatial Population Dynamics

So far, we have presented the functional classes and analyzed the quasispecies from a “static” perspective, that is, by exploring the mutational neighborhood of the master sequence. We now turn to the field to assess which effects the functions defined above have, and at which scale.

Limited diffusion is essential for survival, as increasing the number of random walk-steps per reaction step (to a ratio 3:1), as well as mixing the system, leads to extinction quickly. Nevertheless, at a first glance, the field looks patchy and disorganized. Copies of the master sequence are more or less clustered and separated from parasites, which (as expected) are closer to the regions with empty space. The other units of replications, as well as helpers, stallers and junk, are widespread, with limited apparent spatial clustering (supplementary material and fig. S3, Supplementary Material online. Notice that the system is far from being mixed, as increasing the frequency of random walk-steps per reaction step leads to extinction quickly, and so does mixing the system every time step).

**Fig. 7.—:**
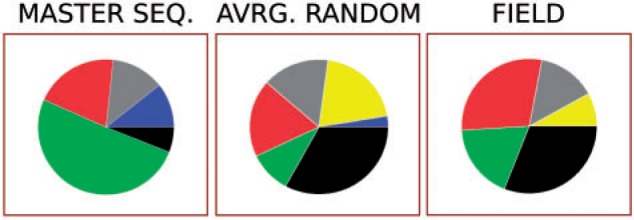
The piecharts represent the mutational neighborhood of left: the master sequence, centre: random units of replication, right: relative abundance of functional classes in the field at one time point (fractions: units of replication 0.31, helpers 0.18, stallers 0.29, junk 0.14, parasites 0.08). The colors in the piecharts are as in figure 4.

**Fig. 8.—:**
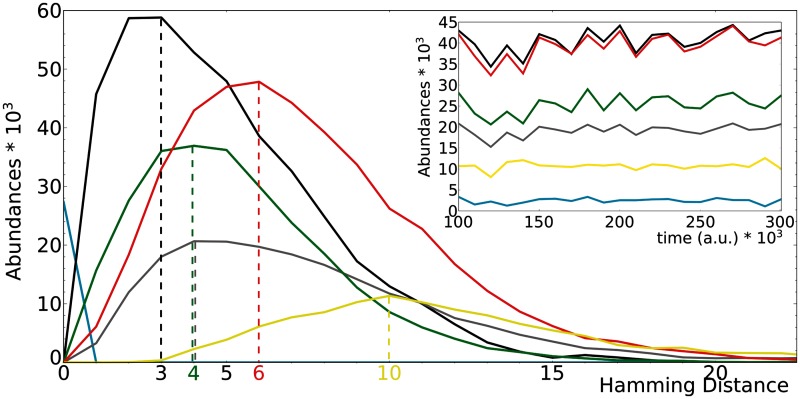
Distribution of the abundances of the functional classes in the field (at one time point) as function of the Hamming Distance of their sequence from the master sequence. The Hamming Distance value is calculated as the minimum between the Hamming Distance of the sequence and the master sequence, and the Hamming Distance of the complementary sequence with the master sequence. Inset: Time plot of the abundances of the various functional classes (the total population is between $100\times 10^{3}$ and $150\times 10^{3}$ individuals). Color coding as in figure 4; in cyan, the master sequence.

The total number of individuals oscillates in time (occupying in total about half of the field); however, as the relative ratio of functional classes remains almost constant (fig. 8), we can study the distribution in the field from a single time point. The individuals at HD = 1 from the master sequence (fig. 8) distribute similarly to the HD = 1 mutants (see fig. 7), the exception being the great abundance of units of replication. The relative fractions of functional classes, however, distribute in a way roughly similar to random (fig. 7).

Units of replication and parasites are selected in the short run (their number is large) because they are viable. Nonetheless, most sequences are nonviable at any given moment: Helpers, and to a lower magnitude, junk, are more frequent at lower Hamming Distances, whereas stallers reach their peak at higher ones. The distribution of parasites has a peak even further in Hamming Distance. Notice that in the field it is possible to discriminate the strand of a hybrid genotype, which can be assigned to helpers or stallers. Figures 8 and 6 share some degree of similarity (until HD = 10) in that helpers decrease with higher Hamming Distances, stallers increase, parasites increase only for HD > 3, and the fraction of junk does not show much variation. Analogously, figure 3 (top) shows that helpers and units of replication are more frequently found close (genotypically) to the master sequence, whereas stallers and parasites arise far from it.

Altogether, there seems to be a correlation between the distributions of mutants of the master sequence and the distribution of functional classes present in the field. To better explain the particular distribution of the functional classes, more information about the behavior of the nonviable phenotypes is needed, which is the concern of the next section.

### The Role of Nonviable Mutants for the Stability of the System

So far, the nonviable phenotypes have been classified, assigned a function and their presence in the field has been shown. It still remains to determine what role they have in terms of ecological stability of the system, especially for what regards helpers and stallers, which is the concern of this section.

#### Helpers

To assess the role of helpers, we exclude them from the field in two ways: Either by removing them, leaving empty space (in which case they still benefit the system in that the empty space becomes a resource), or by turning them into junk (i.e., inert material). In both cases, the system goes extinct quickly. Helpers are crucial for the viability of the whole system.

Because helpers are widespread in the field (supplementary material and fig. S3, Supplementary Material online), parasites benefit from them as well. To investigate the interplay of helpers and parasites for the stability of the system (especially at high mutation rates), we study a simple system of ordinary differential equation (ODE) model. We assume that units of replication (*X*, we ignore the +/− strand difference) can form complexes with other units, helpers (*H*) and parasites (*P*), and the last two can also form complexes. Upon complex formation, new molecules are generated. The mutational products of units of replication are helpers, parasites, and junk (which is inert). We assume that parasites do not mutate, which is justified by their (generally) high neutrality and the lack of helpers in their mutational neighborhood; for simplicity, we do not include junk produced by them, thereby modeling strong parasites. The reaction scheme goes as follows:
(1)
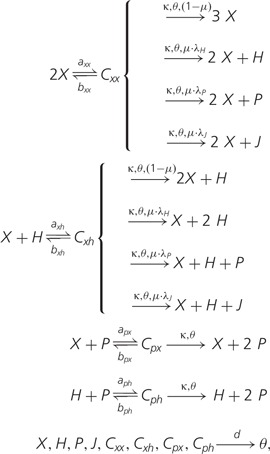

where *a* is the rate of complex formation for the molecules indicated by the subscripts, *b* is the complex dissociation rate, and *C* is a complex between two molecules indicated in the subscript. *κ* is the replication rate (it can be thought of as a polymerization rate), *θ* is a phenomenological term for competition (e.g., resources, such as empty space), *μ* is mutation rate, and *λ* is the fraction of mutants that turn into the class in the subscript. The ODE system from this reaction scheme and a summary of the bifurcation analysis can be found in supplementary material, Supplementary Material online.

**Fig. 9.—:**
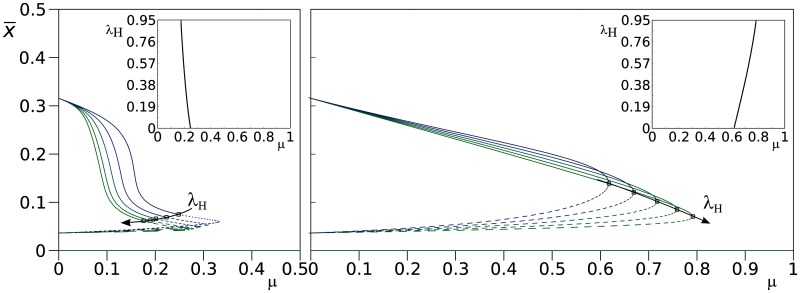
Bifurcation diagrams for the ODE systems with helpers (reaction scheme 1). Left: With parasites. Parameters: $a_{hp}=0.7,a_{xh}=0.7,a_{xp}=0.7,a_{xx}=0.7,d=0.03,\lambda_{P}=0.03,\kappa =1,\theta =1$. Continuous line: stable equilibrium; dashed line: unstable equilibrium; black squares: bifurcation points. The arrow highlights the direction of change for the bifurcation point with progressively increasing $\lambda_{H}$ values ($\lambda_{H}\in \{0,0.25,0.5,0.75,0.95\}$). For simplicity, the Hopf bifurcation is marked rather than the homoclinic bifurcation. However, the two bifurcations are very close. For all parameters combinations, the extinction state $\left(\bar{0}\right)$ is a stable equilibrium. Inset: Two parameter bifurcation plot (*μ* vs. $\lambda_{H}$). Right: Same as above, except parasites are not included in the ODE. The arrow highlights the direction of change of the limit point. Numerical integration and bifurcation analysis performed with GRIND and CONTENT (Kuznetsov 1999; de Boer and Pagie 2005).

In figure 9 (left pane), the steady-state values of the units of replication are plotted against mutation rate (*μ*) for different values of the fraction of mutants that turn into helpers, $\lambda_{H}$ (bifurcation plot). Interestingly, the system is destabilized at lower mutation rates for higher values of $\lambda_{H}$. This effect is entirely due to the presence of parasites and stays the same if the fraction of mutants that turn into parasites ($\lambda_{p}$) is set to zero. To show this, the parasite equation is removed from the ODE system, and a similar bifurcation plot is built (fig. 9, right pane). In contrast with the previous case, increasing the fraction of mutants that turn into helpers ($\lambda_{h}$) makes the system more resistant to mutations (see inset of fig. 9, right pane). The comparison of the two systems shows that in the absence of parasites, helpers stabilize units of replication to a large extent, but it still remains that the system is fragile to parasites: At high mutation rates, the latter can invade and cause extinction (not shown). Conversely, the production of parasites as mutants of units of replication destabilizes the system and leads to extinction at much lower mutation rates, no matter the value of $\lambda_{H}$. We conclude that in the full system helpers are only favorable for the master sequence (and, possibly, for the units of replication very close to it), because of its lack of parasites in both the mutational and the physical neighborhoods. In the next section, we show how the latter is mediated by stallers.

#### Stallers

To investigate the role of stallers (similarly to the case with helpers), we exclude them from the field either by removing them or by replacing them with junk. In both cases the density of the units of replication in the field increases abruptly, and two new lineages evolve: One catalytic and one parasitic, with the system self-organizing into travelling waves. The strategy of units of replication in the new lineage is still “C”-based, whereas parasites are similar in structure to those evolving at lower mutation rates (see Takeuchi and Hogeweg 2008). In the case where stallers are removed from the field, the new catalytic lineage lacks a persisting master sequence, has high neutrality (≈0.3) and few helpers in the mutational neighborhood. An increase in neutrality of units of replication is both a way to cope with high mutation rates and a mechanism to partially weaken the parasitic exploitation, because it increases the variability in the system. In contrast, for the case where stallers are turned to junk, the units of replication are characterized by low neutrality, a moderate amount of helpers, and a large fraction of stallers (tuned to junk). However, the new master sequence evolves so that what is classified as junk behaves partially as stallers. This quasispecies outcompetes the parasitic one, which goes extinct.

That stallers hinder the growth of units of replication (in general) can be seen from the initial, sudden increase of density in the field, when stallers have just been removed. As the quasispecies is highly homogeneous across the field (see fig. 3, top), the increased number of units of replication makes it easier for a parasite to invade (to some extent, further facilitated by the fact that the stallers in their mutational neighborhood are also excluded). In this sense, stallers affect parasites mostly by removing the substrate for their replication (i.e., units of replication), and only to a lesser extent by direct interaction with them. In such conditions, parasites optimize the exploitation of the units of replication, by loosing both 5′-dangling ends while increasing the density of “G” on both 3′-ends. Given the evolution of faster replicating parasites, units of replication that rely on helpers for survival, such as the master sequence, are counterselected and go extinct (as explained above).

Altogether, we conclude that stallers are an intrinsic problem for the system, and that the master sequence of the full model has evolved some “mutational control” over them.

Similarly to the case with helpers, an ODE model is studied to understand the interplay of units of replication, stallers, and parasites. When units of replication (*X*) replicate erroneously, mutants can be junk (*J*), parasites (*P*), or stallers (*S*); the erroneous replication of parasites produces mutants as well, namely junk or stallers. Stallers engage in complex with both parasites and units of replications, but no replication happens. The reaction scheme of the system reads:

(2) $$\begin{array}{l} \text{2 }X\overset{a_{xx}}{\underset{b_{xx}}{⇌}}C_{xx}\{\begin{array}{c} \overset{\kappa ,\theta ,(1-\mu )}{\to }3X\\ \;\overset{\kappa ,\theta ,\mu \cdot \lambda_{S}}{\to }2X+S\\ \;\overset{\kappa ,\theta ,\mu \cdot \lambda_{P}}{\to }2X+P\\ \;\overset{\kappa ,\theta ,\mu \cdot \lambda_{J}}{\to }2X+J \end{array}\\ P+X\overset{a_{px}}{\underset{b_{px}}{⇌}}C_{px}\{\begin{array}{c} \overset{\kappa ,\theta ,(1-\mu )}{\to }2P+X\\ \;\overset{\kappa ,\theta ,\mu \cdot \lambda_{S}}{\to }P+X+S\\ \;\overset{\kappa ,\theta ,\mu \cdot \lambda_{J}}{\to }P+X+J \end{array}\\ X+S\overset{a_{xs}}{\underset{b_{xs}}{⇌}}C_{xs}\\ P+S\overset{a_{ps}}{\underset{b_{ps}}{⇌}}C_{ps}\\ X,S,P,J,C_{xx},C_{px},C_{xs},C_{ps}\overset{d}{\to }\theta , \end{array}$$

where the names of variables and parameters are assigned as above. The ODE system derived from this reaction scheme, as well as a summary of the bifurcation analysis, can be found in supplementary material, Supplementary Material online.

The model formulated here is very similar to those studied in Takeuchi and Hogeweg (2007b). However, the main focus there was on understanding how the interplay of mutation rates with the affinity of the parasites for replicators affected the stability of the system (and in that sense, the comparison with the spatial system was made at a mesoscale level). Here, we are interested in the average behavior of the system at a small scale, where units of replication, stallers and parasites have similar dangling ends, making the rate of the reactions $X+S\overset{a_{xs}}{\to }C_{xs}$ and $P+S\overset{a_{ps}}{\to }C_{ps}$ comparable to $P+X\overset{a_{px}}{\to }C_{px}$ and rather high. The important difference between the master sequence and the other units of replication (at high Hamming Distance) is that the latter has more stallers in the mutational neighborhood than the former, that is, the probability that a staller is produced ($\lambda_{\text{S}}$) is higher.

**Fig. 10.—:**
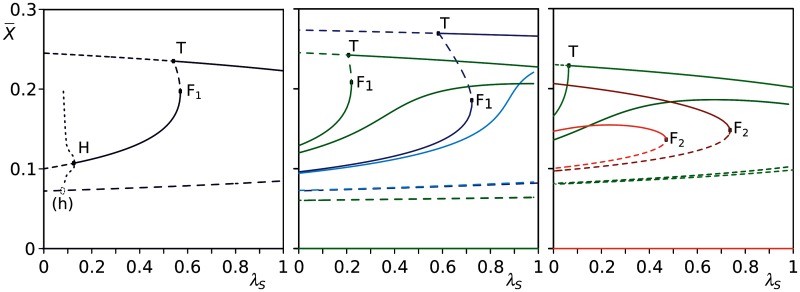
Bifurcation diagrams for the ODE system with stallers (reaction scheme 6). Solid lines: stable equilibria, dashed lines: unstable equilibria, dotted lines: max/min limit cycle, dots: bifurcations ((*h*), estimated location of possible homoclinic bifurcation; *H*, Hopf; *F*_1_ and *F*_2_, fold; *T*, transcritical bifurcation). Left: General figure, parameters: $\lambda_{P}=0,\mu =0.4,a_{ps}=a_{xs}=0.75,a_{xp}=0.8$. Centre: changing *μ* and $\lambda_{P}$ (green: *μ* = 0.3, blue: *μ* = 0.4; dark color: $\lambda_{P}=0$, light color: $\lambda_{P}=0.02$), other parameters: $a_{ps}=a_{xs}=0.7,a_{xp}=0.775$. Right: highest values of *μ* (green: *μ* = 0.45, red: *μ* = 0.52; dark color: $\lambda_{P}=0$, light color: $\lambda_{P}=0.02$), other parameters as before. Parameters common to all three figures: $a_{xx}=0.9,d=0.03,\kappa =1,\Theta =1$. For all parameters combinations, the extinction state $\left(\bar{0}\right)$ is a stable equilibrium.

The system displays a wide range of behaviors. Figure 10 gives an overview of the concentration of units of replication, *X*, as a function of the rate at which stallers are produced, $\lambda_{\text{S}}$, for different combinations of other parameters. For lower mutation rates, in the absence of parasites ($\lambda_{P}=0$), units of replication are minimally hindered by stallers (uppermost line in the left and center pane of fig. 10). However, if the fraction of mutants that turn into stallers is too low ($\lambda_{\text{S}}<\lambda_{\text{S}}(h)$), parasites can invade and lead the system to extinction. Increasing $\lambda_{\text{S}}$ weakens progressively more the exploitation of units of replication by parasites (stable lines in the middle of left and center pane of fig. 10) until the point ($\lambda_{\text{S}}=\lambda_{\text{S}}(F_{1})$) where parasites cannot invade any longer. A similar result holds if parasites are generated as mutants of units of replication (fig. 10, center pane). A large production of stallers at higher mutation rates becomes deleterious for units of replication and may lead the system to extinction ($\lambda_{\text{S}}>\lambda_{\text{S}}(F_{2})$, fig. 10, right pane).

In conclusion, stallers are unavoidable in the full model, especially at higher mutation rates, and they constitute a hinder for units of replication if abundant in the mutational neighborhood, while providing a form of defense against parasites. The master sequence has a limited fraction of them in its mutational neighborhood, which exposes it to the risk of invasion by parasites. However, mutant units of replication have a high fraction of stallers in their mutational neighborhood, which protect the system from parasites by sequestering such units into complex with stallers, or by decreasing the chance that contacts happen between parasites and units of replication. In practice, such strategy favors the master sequence while hindering everybody else in the long run.

### Global Picture of Quasispecies: A Functional Ecosystem

**Fig. 11.—:**
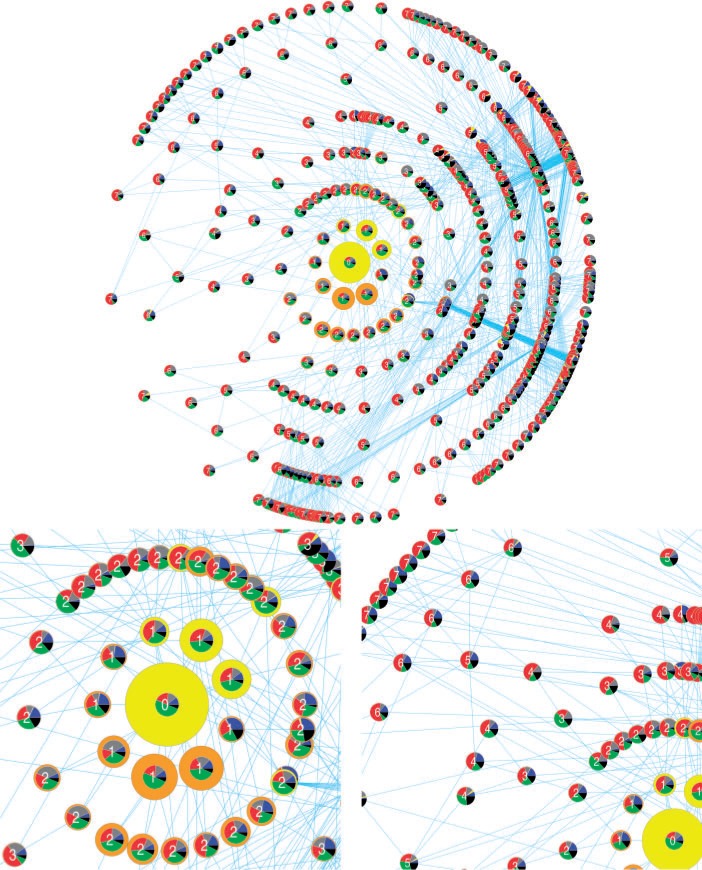
Overview of the units of replication in the quasispecies (top) and close-ups (bottom). Units of replication are generated by following the procedure explained in the caption of figure 4. Each node represents a unit of replication and each edge connects two units of replication if they are at Hamming Distance = 1 from each other. The number in the centre of the node is the Hamming Distance from the master sequence. Each node shows the mutational neighborhood of the unit of replication it represents (the pie chart in the centre, color coding as in fig. 4), and its abundance (the size of the ring around), which is colored according to whether it is a core neutral mutant of the master sequence (in yellow) or not (orange). The radial layout is meant to stress the central role the master sequence has in shaping the rest of the quasispecies. In order to make the figure clearer, units of replication are shown only up to Hamming Distance = 7. The network is visualized with Cytoscape 2.8.

After having elucidated the functional role of nonviable mutants, results can be summed up to present the general picture of the quasispecies. In figure 11 (upper pane), the quasispecies is presented as a network of mutationally adjacent units of replication. The only clearly abundant units of replication are those closest in Hamming Distance to the master sequence (fig. 11, lower left pane): This is understandable because, on the one hand, they are often generated from the master sequence, on the other, and more importantly, their mutational neighborhood is the most similar to that of the master sequence. Upon inspection, it is clear that already at HD = 2 no sequences are as abundant. The abundance of each unit of replication seems to correlate more with the abundance of helpers and with the scarcity of stallers in their mutational neighborhood, rather than with the replication rate of the sequence (e.g., cf. the two units of replication at HD = 1 in orange at the bottom, with the neutral ones at the top, in yellow). The lower right pane of figure 11 shows the progression of mutant units of replication toward the periphery (in genotype space) of the quasispecies. The more Hamming Distance increases, the higher the fraction of stallers in the mutational neighborhood becomes. This explains why units of replication closer to the master sequence are selected (in the short run), as seen in the field (fig. 8), why the peak of the distribution of helpers is one step after (in Hamming Distance) from that of units of replication, and why the peak of the distribution of stallers is at higher Hamming Distances.

Altogether, the evolved quasispecies is under the mutational control of the master sequence, which minimizes the competition with other units of replication by its low degree of neutrality, minimizes the hinder from stallers whereas it maximizes the help received. Other units of replication are selected in the short run provided their mutational neighborhood has a high enough fraction of helpers. However, at higher Hamming Distances the fraction of stallers in their mutational neighborhood makes them effectively nonviable. Helpers have been shown to be necessary for the survival of the whole system, whereas stallers contribute to the global stability of the quasispecies (i.e., the master sequence) by sequestering units of replication and by limiting their accessibility to parasites. No particular selection pressure is found to act on the frequency or the production of inert molecules (junk). In conclusion, the quasispecies behaves functionally like an ecosystem, where different emergent functions are acquired by nonviable sequences and have particular and defined roles.

### Generality of the Results, Steep versus Flat Quasispecies

The initial step to evolve units of replications to higher mutation rates was repeated both here and in Takeuchi and Hogeweg (2008). Including the one described so far, a total of eight units of replication were evolved. These sequences can survive to mutation rates within the range 0.014<μ<0.0165 (see table 1). In most cases (6/8), these sequences exploit a catalytic strategy based on C’s (as the unit of replication described above), and their mutational neighborhood is characterized by a lower degree of neutrality, a higher fraction of helpers and a minimal fraction of parasites. Less frequently (2/8 cases), sequences exploit a catalytic strategy that seems to rely more on A’s than other nucleotides, and, more importantly, they have a higher degree of neutrality and a lower fraction of helpers in their mutational neighborhood, although parasites are still minimized.

**Table 1:** Properties of Units of Replications that Survive at μ≥0.014

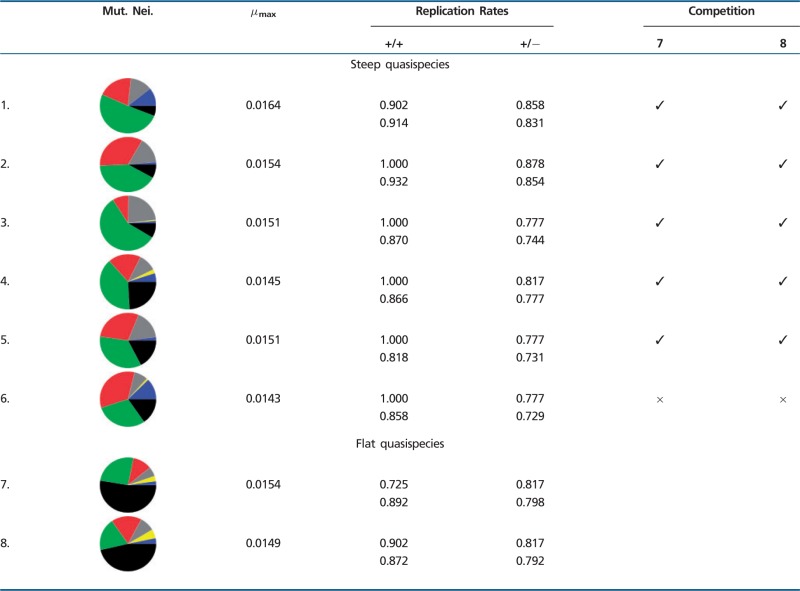

Note.—First column: Pie charts of the HD = 1 mutational neighborhood of the master sequence (for cases 4. and 5. no single master sequence is present, the pie chart is an average of the common ancestors along the line of descent collected every $5\times 10^{4}$ time steps. Analogously for case 6., where the most abundant individuals were collected every $5\times 10^{4}$ time step); Second column: Value of *μ* between the maximum *μ* for which the system does not go extinct and the minimum *μ* for which it does, confidence interval: ± 0.0001; Third and fourth columns: Upper row: Probability of complex formation of the master sequence (or the first common ancestor) with itself, lower row: average probability of complex formation for units of replication within the quasispecies. Left: Catalytic strands with other catalytic strands; Right: With complementary sequences (calculated by sampling $5\times 10^{2}$ units of replication, determining all vs. all probabilities, then averaging); Fifth and sixth columns: Competition experiment with flat quasispecies (7. and 8.). ✓: the steep one outcompetes the flat one, × : vice versa (the experiments are performed by initializing half of the field with one quasispecies and half with other, both taken from runs at sufficiently late time steps).

The units of replication belonging to the steep quasispecies not always display a unique master sequence: In some cases (4., 5., and 6. in table 1), a small group of core neutral mutants with almost identical mutational neighborhood substitutes it. However, the cases that resist to the highest mutation rates do have a unique master sequence (1., 2., and 3. in table 1).

#### Survival of the Flattest

The two sequences with a higher degree of neutrality (7. and 8. in table 1) seem to be a qualitatively different outcome at high mutation rates. Their sequence composition is similar to the units of replication that evolve at lower mutation rates (the “A-catalyst” observed in Takeuchi and Hogeweg 2008). In both cases, the quasispecies have no persisting master sequence, and the sequence variability in the field is high. Moreover, parasites are much more abundant in the field than in the case of steep quasispecies (see supplementary fig. S4, Supplementary Material online). The characteristics of these two quasispecies clearly point to a survival of the flattest effect.

With this in mind, and given that the overall replication rate of steep and flat quasispecies is comparable, we performed all the pairwise competitions between the flat quasispecies and the steep ones. In all cases but one, the steep quasispecies outcompete the flat ones in little time (notice that in the case in which the steep quasispecies is outcompeted, its Error Threshold is very close to the mutation rates used for the competition experiment).

## Discussion

In this study we investigated the eco-evolutionary dynamics at high mutation rates of interacting replicators with an explicitly defined genotype (RNA-like sequences), and a phenotype that depends on a complex genotype-to-phenotype map (RNA folding). We show that a quasispecies evolves which is steep and in which emergent functionalities are associated to nonviable individuals, and critically contribute to the overall stability and long-term persistence of the system. The information on the structure of the functional ecosystem is coded on the master sequence, and stochastically decoded through mutations.

A key feature of the model is that neither an interaction structure is preconceived, nor it is predefined which sequences are most fit (i.e., no fitness function is imposed on the system). All that is implemented are simple “chemistry” rules, that is, sequence recognition for complex formation, which selection may exploit. Both complex formation and replication are local processes, which means that individuals interact mostly with identical copies of themselves as well as, inevitably, with their close mutants. This implies that interactions, when happening, are usually strong (i.e., if the appropriate dangling ends are present, the probability of complex formation and replication is high). Colocalization and strong interaction are at the basis of the observed evolutionary (positive) feedback: Units of replication are selected (at least in the short run) if their close mutants replicate them more than the mutants themselves get replicated. This happens in two ways: Close mutants (both in genotype and in physical space) are exploited for giving replication without being able to replicate (helpers), further away mutants (stallers) block competing units of replication and parasites. So, although genotypically very close, these sequences are phenotypically and functionally different. Altogether, the evolutionary structuring of this mutational process establishes the functional linkages within the quasispecies. The extent of the mutational control the master sequence reaches is surprisingly deep (Hamming Distance ≈10, where it becomes indistinguishable from random).

This model was initially studied in Takeuchi and Hogeweg (2008), where it was argued that the evolution of ecological complexity is limited at greater mutation rates, where a single, invariant quasispecies persists. Here we see that low diversity at sequence level enables a specific mutational neighborhood and therewith a large functional diversity. The evolution of new lineages is prevented by the structured mutational neighborhood at high mutation rates. At low mutation rates, the amount of functionally different mutants generated within the quasipecies is reduced, and separate lineages, for example, parasites, readily evolve.

In conclusion, mutation rate “automatically” classifies different eco-evolutionary dynamic regimes, even though the genotype–phenotype-interaction map is identical.

### 

#### One-to-Many Genotype-to-Phenotype Map

The outcome of the evolutionary dynamics consists of a sequence, which stores the information for the entire functional ecosystem. Functions are unfolded through minimal variations (mutations). In our model, substitutions are the only mechanism that can allow the spatial and functional control required to evolve the complex interaction structure observed, hence the need for high mutation rates. Altogether, this represents an example of a one-to-many genotype-to-phenotype map.

Other mechanisms to achieve multiple functionalities with limited coding resources (e.g., in proximity of the Information Threshold) have been explored in silico by Hogeweg and Hesper (1992), and more recently within the context of RNA folding in, for example, Ancel and Fontana (2000), Fontana (2002), de Boer and Hogeweg (2012), and de Boer (2012) (the phenomenon occurs frequently in vivo [Trifonov 1989]; see Tuck and Tollervey [2011] for a recent review about RNAs). Although in those models the evolution of a one-to-many genotype-to-phenotype map was the object of study, in our model it was completely unexpected. Similarly to de Boer and Hogeweg (2012), our results make the point that mutation rate should be interpreted as a structural degree of freedom rather than only a limiting factor to information accumulation.

#### Steep Versus Flat Quasispecies

From a quasispecies-theoretical perspective, evolution exploits the very defining feature of quasispecies sensu strictu, namely, that mutations happen often and surely. In our models, this produces an interaction structure. In combination with a complex genotype-to-phenotype map, some control over these mutants can emerge.

This cannot happen if the only selectable trait is replication rate: When mutations happen frequently (with large enough populations), populations evolving on neutral networks automatically increase their mutational robustness (Van Nimwegen et al. 1999). Quasispecies models that display the survival of the flattest effect (originally Schuster and Swetina [1988], later emphasized by Wilke et al. [2001]) suffer from the same limitation. Indeed, the spatially extended versions of these models show that the range of mutation rates in which flat quasispecies outcompete steeper ones is larger than in the well-mixed case (Sardanyés et al. 2008). Multiple selection steps at different life stages can lead to favoring antirobust individuals (as they purge deleterious mutations faster), but this is not the case at higher mutation rates (Archetti 2009).

In contrast, our model shows that the evolution of an interaction structure and the lack of neutrality are closely linked. This explains why the increase in mutational robustness is not observed in most cases, and why, for the cases in which flatter solutions evolve, they are most often outcompeted by quasispecies with a highly structured mutational neighborhood. In other words, survival of the flattest does not happen. Sequences in flat quasispecies are, to some extent, under the same selection pressure as the steep ones, that is, selection for a structured mutational neighborhood (which can be seen from the moderate fraction of helpers and the low fraction of parasites in their mutational neighborhood, in table 1). In this sense, it seems that flat quasispecies are somewhat deregulated solutions in comparison to the steep ones. Nonetheless, flatter quasispecies retain a high maximum replication rate, comparable to that of steep solutions (in contrast to Elena et al. 2007), whereas the average replication rate is even higher (in table 1 the average replication rate is calculated taking into account only units of replication, and not nonviable individuals). This means that higher neutrality in the quasispecies does not come about due to a less effective selection pressure, as is the case in Krakauer and Plotkin (2002) as well as in the Royal Road Genetic Algorithm (Mitchell et al. 1992; van Nimwegen and Crutchfield 2000). The flat quasispecies are outcompeted because they lack the (evolved) properties of the steep ones, namely, a structured mutational neighborhood which establishes a functional linkage within the quasispecies.

It has been shown (Huynen 1993) that in predator–prey dynamics with RNA-like organisms (in which the prey is not eaten if it is not “recognized,” i.e., if the predator’s phenotype does not match that of the prey), populations evolve to steeper regions of the phenotype landscape and the increase in mutational robustness does not happen. Selection acts to rapidly change phenotypes so that individuals that can change the fastest (i.e., those that are in the least neutral portion of the neutral network for a given secondary structure) will have an advantage.

The evolutionary dynamics in that model is very different from ours. In that case it is Red Queen dynamics (Van Valen 1973), whereas in our case a steady-state-like solution is evolved (moreover we do not preconceive any interaction structure). However both models make the point that interacting individuals characterized by a phenotype determined with a complex map (e.g., RNA folding) may evolve to lower degrees of mutational robustness at high mutation rates, as these solutions are more versatile.

#### The Effect of Lethal Mutants

In our model (and conceivably with RNA in general), the loss of viability as a result of mutations manifests at the phenotype level, when the sequence has already been generated. This means that lethal phenotypes (in a broad sense of the word) do not die the moment they are born (which is, instead, the customary modeling approach in quasispecies theory, for example, in Takeuchi and Hogeweg [2007a], Kirakosyan et al. [2010], but not in Tejero et al. [2010]), whereas it still remains that the fitness advantage of the individual that produced them is infinite. The consequences of this (in our model) are important: 1) Viable individuals evolve to increase the fraction of lethal (nonviable) sequences in their mutational neighborhood, with the latter acquiring novel functionalities; and 2) the exploitation of nonviable mutants by units of replication to outcompete other viable individuals becomes more extreme the higher the mutation rates. A remarkable side effect is that neither delocalization nor an Error Threshold sensu strictu happens. For higher mutation rates, the quasispecies hits an extinction threshold when the viable individuals do not produce enough viable offspring.

In conclusion, the presence or absence of delocalization depends on the evolutionary dynamics of the quasispecies. This provides a counter example to the claim made in Holmes (2010) that the “extinction threshold” does not rely on quasispecies dynamics, although our model is obviously not explicitly meant to address the quasispecies dynamics of RNA viruses.

#### Evolution and Persistence of Diversity

Since the original work on the hypercycle (Eigen and Schuster 1978), the problem posed by the Information Threshold has often been addressed as the problem of maintaining (functional) diversity, that is, in terms of persistence of independent lineage (e.g., Boerlijst and Hogeweg 1991; Szathmáry 1991; Happel and Stadler 1998) or evolving it (e.g., Hogeweg 1994).

It was recently shown that genotypic diversity could be maintained if RNA replicators exploited different nucleotide compositions (Szilágyi et al. 2013), although in that case coexistence seems to be very sensitive to single substitutions. In Ma and Hu (2012), a system of functional molecules which cooperate to perform the various steps needed for (protocell) self-replication could spread without loosing diversity. In Könnyű and Czárán (2013) a large number of different species are shown to coexist in a spatially extended system if they are all necessary to the (local) production of resources they all (locally) exploit, even if parasites evolve (depending on the diffusion rates of resources and of replicators). However, (to the best of our knowledge) the evolutionary maintenance of the metabolic diversity is unclear. Nevertheless, within the framework, parasites were shown to acquire replicase activity, thus increasing the overall complexity of the system (Könnyű et al. 2008). The stabilization of replicators/parasites interactions in spatially extended systems is mediated by spatial pattern formation. The eco-evolutionary dynamics of such systems have been studied in depth in Takeuchi and Hogeweg (2009), where it was shown that travelling waves constitute a higher level of selection than that of individual replicators and parasites. In de Boer and Hogeweg (2010), spatial pattern formation mediated the evolution of an ecosystem based information-processing system at high mutation rates. In our case, differently functional individuals and their interactions are emergent phenomena. However, different functions are performed by similar genotypes; hence, diversity is phenotypic rather than genotypic.

#### Division of Labor

From a more general biological perspective, the eco-evolutionary dynamics presented here represent a form of (partial) reproductive division of labor. The evolution of division of labor in an RNA-like system, from a self-replicating molecule to a transcription-like mechanism (i.e., with templates and polymerases), has been shown to evolve because it confers an increased resistance to parasites (Takeuchi et al. 2011). In this respect, we present a different mechanism to achieve this. What we observe is 2-fold: 1) A line of descent of (almost) identical genotypes (the master sequences) carrying the information for their own survival, as well as that for the functions and spatial organization of the mutants and 2) a multitude of mostly nonviable genotypes very similar to those along the line of descent, with very different phenotypes, which come from and aid the further propagation of the master sequences. In this sense, our results are reminiscent of the reproductive division of labor in social insects or that of germline and soma in (possibly early evolutionary stages of) developmental processes, although in those cases regulation instead of mutation underlies the differentiation process.

## Conclusions

Although the details of the results presented here are specific to the arbitrary chemistry implemented, we maintain that the conclusions are general in that:

- A quasispecies evolves which behaves like an ecosystem.
- The emergent functions are carried out by nonviable sequences.
- It constitutes an individually coded, but stochastically decoded ecosystem based solution.
- It exploits the frequently arising mutations by evolving to regions of the genotype-to-map where small genotypic change produces large phenotypic differences.

## Supplementary Material

Supplementary material and figures S1–S4 are available at *Genome Biology and Evolution* online (http://www.gbe.oxfordjournals.org/).

Supplementary Data

## References

[evu150-B1] Ancel LW, Fontana W (2000). Plasticity, evolvability, and modularity in RNA. J Exp Zool..

[evu150-B2] Archetti M (2009). Survival of the steepest: hypersensitivity to mutations as an adaptation to soft selection. J Evol Biol..

[evu150-B3] Boerlijst MC, Hogeweg P (1991). Spiral wave structure in pre-biotic evolution: hypercycles stable against parasites. Physica D.

[evu150-B4] de Boer FK (2012). Coding flexibility or how evolution shapes the structure and complexity of coding [PhD thesis]. Utrecht University, Theoretical Biology and Bioinformatics..

[evu150-B5] de Boer FK, Hogeweg P (2010). Eco-evolutionary dynamics, coding structure and the information threshold. BMC Evol Biol..

[evu150-B6] de Boer FK, Hogeweg P (2012). Less can be more: RNA-adapters may enhance coding capacity of replicators. PLoS One.

[evu150-B7] http://bioinformatics.bio.uu.nl/rdb/software.html.

[evu150-B8] de Boer RJ, Staritsky AD (2000). http://bioinformatics.bio.uu.nl/rdb/software.html.

[evu150-B9] Eigen M (1971). Selforganization of matter and the evolution of biological macromolecules. Naturwissenschaften.

[evu150-B10] Eigen M, Schuster P (1978). The hypercycle. Naturwissenschaften.

[evu150-B11] Elena SF, Wilke CO, Ofria C, Lenski RE (2007). Effects of population size and mutation rate on the evolution of mutational robustness. Evolution.

[evu150-B12] Fontana W (2002). Modelling ‘evo-devo’ with RNA. BioEssays.

[evu150-B13] Fontana W, Konings DA, Stadler PF, Schuster P (1993). Statistics of RNA secondary structures. Biopolymers.

[evu150-B14] Happel R, Stadler PF (1998). The evolution of diversity in replicator networks. J Theor Biol..

[evu150-B15] Hermisson J, Redner O, Wagner H, Baake E (2002). Mutation–selection balance: ancestry, load, and maximum principle. Theor Popul Biol..

[evu150-B17] Hogeweg P (1994). Multilevel evolution: replicators and the evolution of diversity. Physica D.

[evu150-B18] Hogeweg P, Hesper B (1992). Evolutionary dynamics and the coding structure of sequences: multiple coding as a consequence of crossover and high mutation rates. Comput Chem..

[evu150-B19] Hogeweg P, Takeuchi N (2003). Multilevel selection in models of prebiotic evolution: compartments and spatial self-organization. Orig Life Evol Biosph..

[evu150-B20] Holmes EC (2010). The RNA virus quasispecies: fact or fiction?. J Mol Biol..

[evu150-B21] Huynen M (1993).

[evu150-B22] Huynen MA (1996). Exploring phenotype space through neutral evolution. J Mol Evol..

[evu150-B23] Huynen MA, Konings DA, Hogeweg P (1993). Multiple coding and the evolutionary properties of RNA secondary structure. J Theor Biol..

[evu150-B24] Kirakosyan Z, Saakian DB, Hu C-K (2010). Evolution models with lethal mutations on symmetric or random fitness landscapes. Phys Rev E..

[evu150-B25] Könnyű B, Czárán T (2013). Spatial aspects of prebiotic replicator coexistence and community stability in a surface-bound RNA world model. BMC Evol Biol..

[evu150-B26] Könnyű B, Czárán T, Szathmáry E (2008). Prebiotic replicase evolution in a surface-bound metabolic system: parasites as a source of adaptive evolution. BMC Evol Biol..

[evu150-B27] Krakauer DC, Plotkin JB (2002). Redundancy, antiredundancy, and the robustness of genomes. Proc Natl Acad Sci U S A..

[evu150-B28] Kuznetsov YA (1999). www.staff.science.uu.nl/k~ouzn101/CONTENT/.

[evu150-B29] Letunic I, Bork P (2011). Interactive tree of life v2: online annotation and display of phylogenetic trees made easy. Nucleic Acids Res..

[evu150-B30] Ma W, Hu J (2012). Computer simulation on the cooperation of functional molecules during the early stages of evolution. PLoS One.

[evu150-B31] Mitchell M, Forrest S, Holland JH (1992). The royal road for genetic algorithms: fitness landscapes and GA performance. Proceedings of the First European Conference on Artificial Life.

[evu150-B32] Sardanyés J, Elena SF, Solé RV (2008). Simple quasispecies models for the survival-of-the-flattest effect: the role of space. J Theor Biol..

[evu150-B33] Schuster P, Fontana W, Stadler PF, Hofacker IL (1994). From sequences to shapes and back: a case study in RNA secondary structures. Proc R Soc Lond B Biol Sci..

[evu150-B34] Schuster P, Swetina J (1988). Stationary mutant distributions and evolutionary optimization. Bull Math Biol..

[evu150-B35] Smoot ME, Ono K, Ruscheinski J, Wang P-L, Ideker T (2011). Cytoscape 2.8: new features for data integration and network visualization. Bioinformatics.

[evu150-B36] Szathmáry E (1991). Simple growth laws and selection consequences. Trends Ecol Evol..

[evu150-B37] Szathmáry E, Demeter L (1987). Group selection of early replicators and the origin of life. J Theor Biol..

[evu150-B38] Szilágyi A, Zachar I, Szathmáry E (2013). Gause’s principle and the effect of resource partitioning on the dynamical coexistence of replicating templates. PLoS Comput Biol..

[evu150-B39] Takeuchi N, Hogeweg P (2007a). Error-threshold exists in fitness landscapes with lethal mutants. BMC Evol Biol..

[evu150-B40] Takeuchi N, Hogeweg P (2007b). The role of complex formation and deleterious mutations for the stability of RNA-like replicator systems. J Mol Evol..

[evu150-B41] Takeuchi N, Hogeweg P (2008). Evolution of complexity in RNA-like replicator systems. Biol Direct..

[evu150-B42] Takeuchi N, Hogeweg P (2009). Multilevel selection in models of prebiotic evolution II: a direct comparison of compartmentalization and spatial self-organization. PLoS Comput Biol..

[evu150-B43] Takeuchi N, Hogeweg P, Koonin EV (2011). On the origin of DNA genomes: evolution of the division of labor between template and catalyst in model replicator systems. PLoS Comput Biol..

[evu150-B44] Tejero H, Marín A, Montero F (2010). Effect of lethality on the extinction and on the error threshold of quasispecies. J Theor Biol..

[evu150-B45] Trifonov EN (1989). The multiple codes of nucleotide sequences. Bull Math Biol..

[evu150-B46] Tuck AC, Tollervey D (2011). RNA in pieces. Trends Genet..

[evu150-B47] van Nimwegen E, Crutchfield JP (2000). Optimizing epochal evolutionary search: population-size independent theory. Comput Meth Appl Mech Eng..

[evu150-B48] Van Nimwegen E, Crutchfield JP, Huynen M (1999). Neutral evolution of mutational robustness. Proc Natl Acad Sci U S A..

[evu150-B49] Van Valen L (1973). A new evolutionary law. Evol Theor..

[evu150-B50] Wilke CO, Wang JL, Ofria C, Lenski RE, Adami C (2001). Evolution of digital organisms at high mutation rates leads to survival of the flattest. Nature.

