# Supplementary material for: Evolution of Functional Diversification within Quasispecies
Source: Genome Biol Evol. 2014 Jun 22;6(8):1990–2007. doi: 10.1093/gbe/evu150 (PMC4159002; doi:10.1093/gbe/evu150)
Supplement: Supplementary Data [file supp_6_8_1990__index.html]

Evolution of Functional Diversification within Quasispecies — Supplementary Data 

# Evolution of Functional Diversification within Quasispecies

## Supplementary Data

files

**Files in this Data Supplement:**

- Supplementary Data - pdf file
