# Supplementary material for: Evolution of Functional Diversification within Quasispecies
Source: Genome Biol Evol. 2014 Jun 22;6(8):1990–2007. doi: 10.1093/gbe/evu150 (PMC4159002; doi:10.1093/gbe/evu150)
Supplement: Supplementary Data [file supp_evu150_shape_quasisp_supplementary_material.pdf]

Supplementary Material of: Structure and Evolution of Quasispecies in Interacting Replicators

Enrico Sandro Colizzi and Paulien Hogeweg

July 23, 2014

1 Supplementary material

1.1 Global distribution of functional classes in the field vs. genotype space

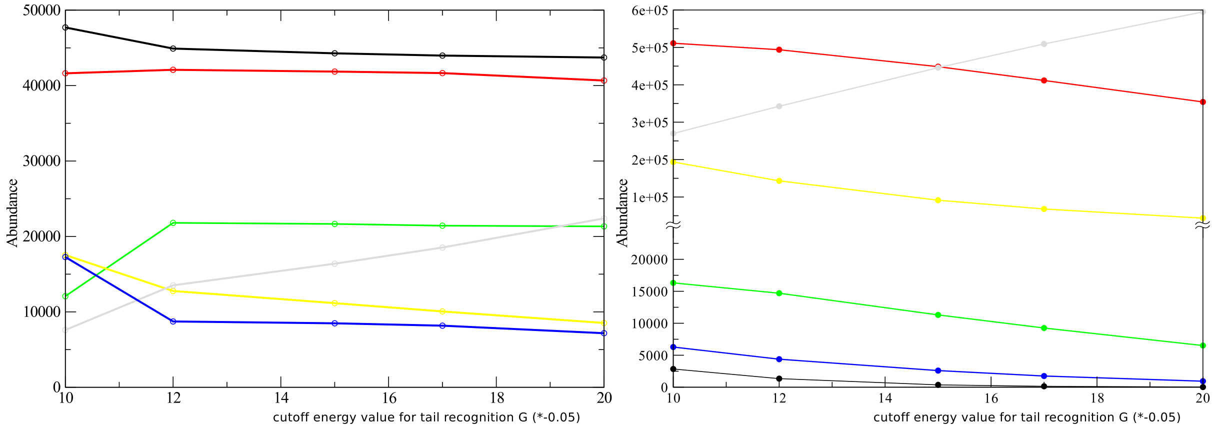

Figure 1: (In)sensitivity of functional classes to the cutoff parameter. Global distribution of functional classes in the CA (left) and in entire genotype space (right). The different abundances in functional classes are shown as function of the cutoff score used to determine presence/absence of dangling ends, in the main text the value corresponding to 15 is used as default (see Models and Methods). Left pane: at one time point of a standard simulation (as described in the main text), all sequences are collected to produce the figure. Right pane: 10^6 random sequences are generated for the figure. Notice that the scale of the two y axis is different.

1.2 Evolution to high mutation rates

As mentioned in the main text, initializing the system at high mutation rates ($\mu = 0.014$ or higher) with randomly generated units of replication always leads to a quick extinction. Here, results are reported about the evolutionary run that leads to sequences able to sustain high mutation rates. A homogeneous population of units of replication is used to initialise the system. The mutation rate (μ) assigned is sligthly greater than zero. μ determines the fidelity by which a unit of replication copies another sequence. When mutations occur, μ is changed as well, by adding a tiny but strictly positive number. An alternative method consists of imposing on all sequences an external mutation rate and setting it to progessively higher values (in little steps). Results do not differ more between runs wich implements different methods than they differ for two runs with the same method. In Fig. 2, one such pre-evolution runs is shown. The distribution of mutation rates is plotted in time for both Units of replication (red) and Parasites (black).

During the pre-evolution step, we distinguish three time-interval groups, on the basis of the dynamic regime the system displays. These three intervals can be characterized by the mutation rates of the individual lineages present during such interval. Patterns are distinguished by clustering the sequences and analyzing their phenotypes, which, as will become clear in the following, corresponds to characterize their ecological role. The three time periods (in arbitrary time units) are 1) Red Queen dynamics: $0 < time < 600 * 10^3$, 2) Multi-species dynamics $600 * 10^3 < time < 1300 * 10^3$, 3) Single (quasi-)species dynamics $time > 1300 * 10^3$. Time boundaries are somewhat arbitrary, as it is not possible to uniquely locate in time when the system changes dynamic regime.

Red Queen dynamics For the first $600 * 10^3$ time steps, the average mutation rate for the catalytic sequences is low ($0 < \mu < 0.003$). As can be seen from Fig. 2 (black box), at time $500 * 10^3$ three different lineages of Units of replication coexist with a parasitic one. All of them can be characterized by a particular nucleotide preference on the 5' dangling end of the catalytic strand (depicted in the figure), or, for the Parasite, the 3' dangling end. All the catalytic lineages have about the same mutation rate, and the variability in the field is limited (see the sequence Logo's). However, the system is not stable and these lineages are not persisting, with the exception of the unit of replication class characterized by 'C' nucleotide. Since Parasites descend from Units of replication, they are assigned a mutation rate, which they inherit generation after generation. Such parameter does not have any actual use for Parasites, because they are not catalytic, but it still mutates with the same algorithm by which a μ assigned to a functional molecule would. Therefore, given that mutation rate is biased to only increase, the exponentially increasing curve of the parasitic branch represents drift. Altogether, the evolutionary dynamics seems to be dominated by a Red Queen-like effect [Van Valen, 1973], with different catalytic strategies rapidly changing in response to the arise of parasitic lineages.

Multi-species dynamics In the second time period, between $600 * 10^3$ and $1300 * 10^3$ time steps, the nucleotide preferences in the lineages become more predictable. When four species are present, the 5' dangling end of all units of replication are composed either by a majority of 'C' or 'A', while the parasites have 3' dangling ends composed either by 'G' or by 'U's. The C-unit of replications are exploited by G-parasites, while the A-units are exploited by the U-parasites (Fig. 2 red box). In the CA this gives rise to mutually invading travelling waves, where each catalyst grows in the empty space left by the parasite it is not exploited by. When the A-units of replication reach high mutation rates, first the U-parasite goes extinct, then the A-units disappear. The C-units maintain a much lower mutation rate and, since the G-parasite is still present, this opens a niche for a new A-unit of replication to evolve, quickly increase the mutation rate, reach the Error Threshold and disappear again. The cycle of evolution and extinction of the A-catalytic lineage repeats a few times. This can be explained as follows: while the C-unit/G-parasite system is present, mutations that turn the C nucleotide preference to the A nucleotide are selected, as they allow a progressively lower exploitation by the G-parasite. However, since mutation rate is biased to increase upon substitutions, the same process that drives the evolution of an A-lineage, also drives it quickly to hit its Error Threshold and make it disappear, opening the niche again for a new A-lineage. However, when mutation rate becomes sufficiently high for the C-unit of replication, new A-catalytic lineages cannot appear any longer and only two species coexist (Fig. 2 blue box).

Single (quasi-)species dynamics In the last time period ($time > 1300 * 10^3$) mutation rate is greater than 0.012, and the system is characterized by a single quasispecies. The structure, the selection pressures and the survival mechanism of this quasispecies are the concern of the main text of the paper.

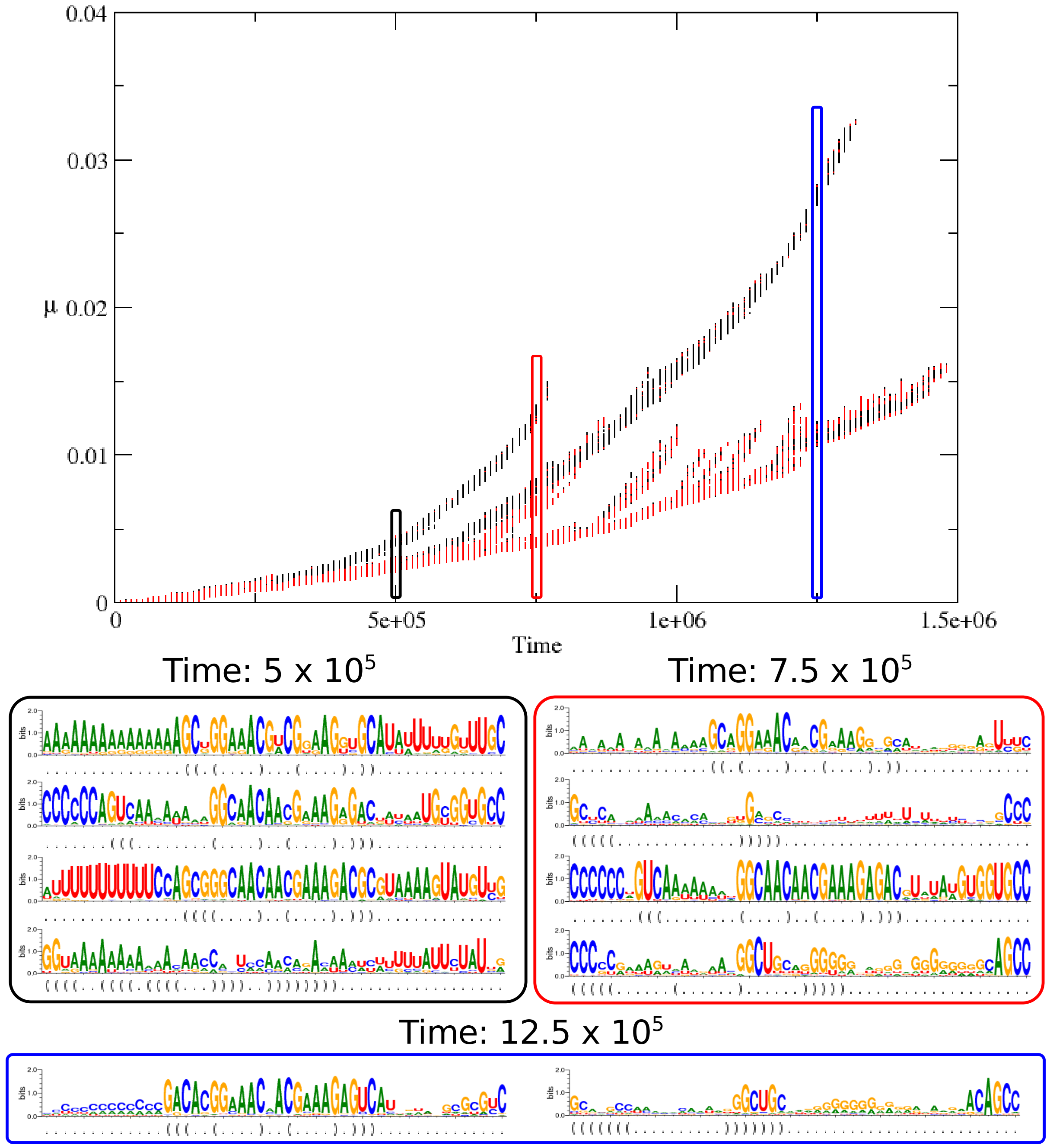

Figure 2: Distribution of mutation rates in time and species composition at different time points. Top pane: every 10^4 timesteps, the mutation rate of each sequence is collected and plotted. Red: catalytic sequences, black: non catalytic sequences. At three different time points, corresponding to different dynamic regimes, a random sample of sequences of the same strand (chosen so that the units of replication display the catalytic one) are clustered and the sequence logo is obtained for each quasispecies (see caption of Fig. 3 in the main text).

1.3 Snapshot of the field for the quasispecies described in the main text

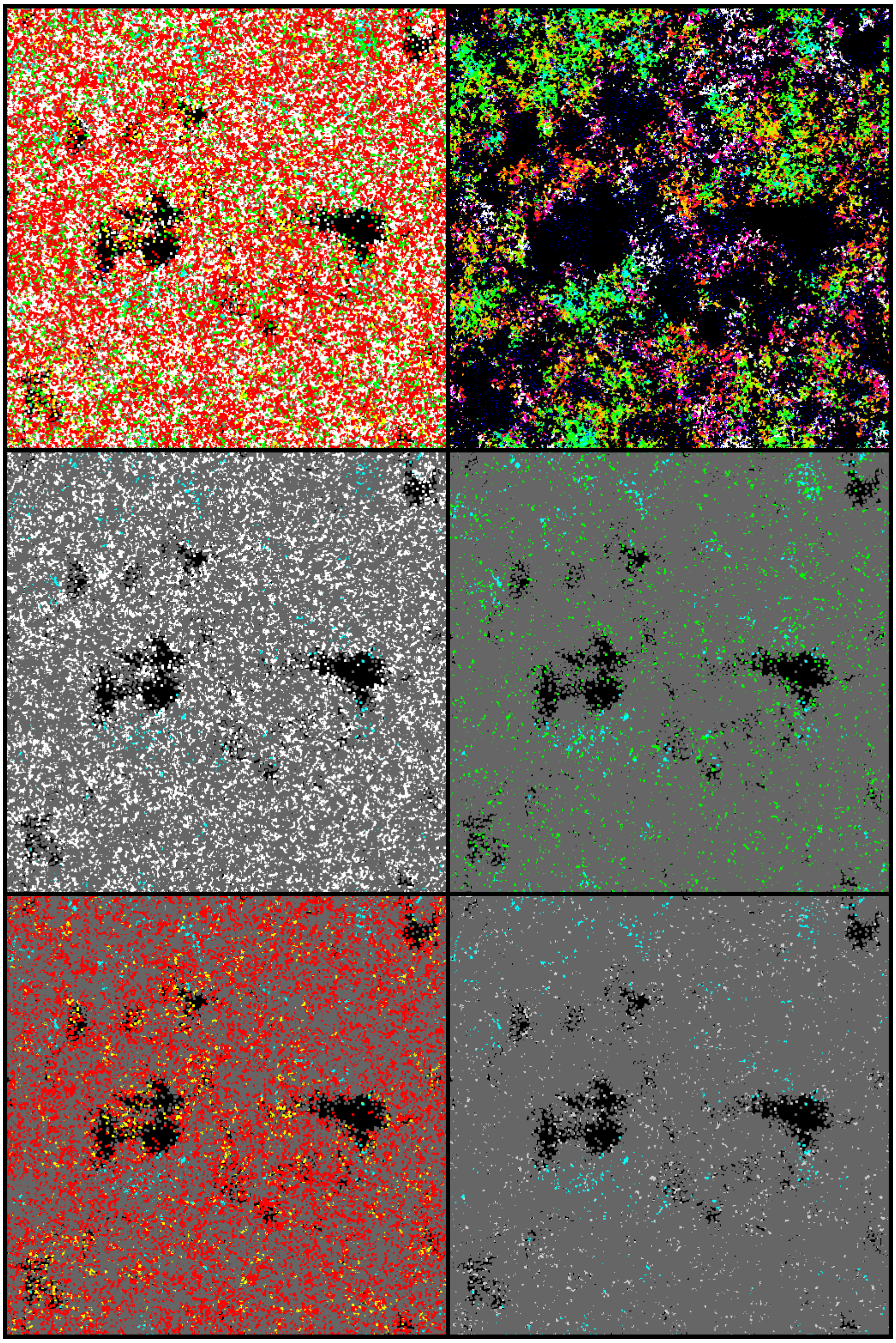

Figure 3: A snapshot of the field: all figures are at the same time point, but coloured differently for each functional class. **Upper-left**: the full field; colours as in the main text. **Upper-right**: The Hamming Distance of all sequences from the master sequence (cyan) is depicted. The colours follow the gradient cyan-green-yellow-red-magenta, corresponding to Hamming Distance 0 to 7. In white, Hamming Distance > 7. Blue: parasites. For all that follows: copies of the master sequence are in cyan, all the classes not explicitely mentioned are in dark grey, background is in black. **Centre-left**: units of replication (white). **Centre-right**: helpers (green). **Bottom-left**: stallers (red), parasites (yellow). **Bottom-right**: junk (light grey). Colouring is as follows: for each cell the functional classes in the Moore neighbourhood are determined (excluding empty cells, except in the upper right figure), and the whole neighbourhood is coloured according to the (functional or Hamming Distance) class in the majority. Notice that the density in the field seems higher than what it actually is.

1.4 ODE system with helpers

The ODE system derived here follows the reaction scheme presented in the main text (reaction scheme 1). For all second order reactions, mass action is assumed:

$$
\begin{aligned}
\dot{x} &= -2a_{xx}x^2 + (2b_{xx} + 3\kappa\theta(1-\mu) + 2\kappa\theta\mu)c_{xx} \\
&\quad -a_{xh}xh + (b_{xh} + 2\kappa\theta(1-\mu) + \kappa\theta\mu)c_{xh} \\
&\quad -a_{px}px + (b_{px} + \kappa\theta)c_{px} - dx \\
\dot{h} &= \kappa\theta\mu\lambda_h c_{xx} - a_{xh}xh + (b_{xh} + \kappa\theta(1-\mu) + \kappa\theta\mu(1+\lambda_h))c_{xh} \\
&\quad -a_{ph}ph + (b_{ph} + \kappa\theta)c_{ph} - dh \\
\dot{p} &= \kappa\theta\mu\lambda_p c_{xx} + \kappa\theta\mu\lambda_p c_{xh} - a_{px}px + (b_{px} + 2\kappa\theta)c_{px} \\
&\quad -a_{ph}ph + (b_{ph} + 2\kappa\theta)c_{ph} - dp \\
\dot{j} &= \kappa\theta\mu\lambda_j c_{xx} + \kappa\theta\mu\lambda_j c_{xh} - dj \\
\dot{c}_{xx} &= a_{xx}x^2 + b_{xx}c_{xx} - \kappa\theta c_{xx} - dc_{xx} \\
\dot{c}_{xh} &= a_{xh}xh + b_{xh}c_{xh} - \kappa\theta c_{xh} - dc_{xh} \\
\dot{c}_{px} &= a_{px}px + b_{px}c_{px} - \kappa\theta c_{px} - dc_{px} \\
\dot{c}_{ph} &= a_{ph}ph + b_{ph}c_{ph} - \kappa\theta c_{ph} - dc_{ph}
\end{aligned}
$$

where $\theta = 1 - (x + h + p + j + c_{xx} + c_{xh} + c_{px} + c_{ph})/\Theta$, $\lambda_h + \lambda_p + \lambda_j = 1$, $b = 1 - a$ and $\kappa = 1$. For all μ, $(\vec{0})$ is a stable equilibrium. Progressively increasing μ, the non trivial, stable equilibrium (solid line in Fig. 9) is at first a fixed point, then a supercritical Hopf bifurcation occurs and a stable limit cycle appears. Further increasing μ, the limit cycle disappears (most likely via a homoclinic bifurcation, when the minimum of the limit cycle touches the line of unstable equilibria) and the only stable equilibrium remaining is $(\vec{0})$, i.e. the system goes extinct (but notice that the difference between the Hopf bifurcation and the homoclinic bifurcation is always very small). Different fraction of mutants turning into helpers λ_H change the bifurcation plot quantitatively. Removing parasites from all the equations (Fig. 9, right pane), changes the bifurcation plot both quantitatively and qualitatively in that both Hopf and homoclininc bifurcations disappear and extinction at high μ is now mediated by a fold bifurcation.

1.5 ODE system with stallers

Analagously to the case with helpers, the ODE system derived for stallers (from the reaction scheme 2) reads:

$$
\begin{aligned}
\dot{x} &= -2a_{xx}x^2 + (2b_{xx} + 3\kappa\theta(1-\mu) + 2\kappa\theta\mu)c_{xx} \\
&\quad -a_{px}px + (b_{px} + \kappa\theta)c_{px} - a_{xs}xs + b_{xs}c_{xs} - dx \\
\dot{s} &= \kappa\theta\mu\lambda_s(c_{xx} + c_{xp}) - a_{xs}xs + b_{xs}c_{xs} - a_{ps}ps + b_{ps}c_{ps} - ds \\
\dot{p} &= \kappa\theta\mu\lambda_p c_{xx} - a_{px}px + (b_{px} + 2\kappa\theta(1-\mu) + \kappa\theta\mu)c_{px} \\
&\quad -a_{ps}ps + b_{ps}c_{ps} - dp \\
\dot{j} &= \kappa\theta\mu\lambda_j(c_{xx} + c_{xp}) - dj \\
\dot{c}_{xx} &= a_{xx}x^2 + b_{xx}c_{xx} - \kappa\theta c_{xx} - dc_{xx} \\
\dot{c}_{xs} &= a_{xs}xs + b_{xs}c_{xs} - dc_{xs} \\
\dot{c}_{px} &= a_{px}px + b_{px}c_{px} - \kappa\theta c_{px} - dc_{px} \\
\dot{c}_{ps} &= a_{ps}ps + b_{ps}c_{ps} - dc_{ps}
\end{aligned}
$$

where $\theta = 1 - (x + s + p + j + c_{xx} + c_{xs} + c_{px} + c_{ps})/\Theta$, $\lambda_S + \lambda_P + \lambda_J = 1$, $b = 1 - a$ and $\kappa = 1$. For all parameters combinations, the extinction state $((\vec{0}))$ is a stable equilibrium.

Higher a_{**} values, $\lambda_P = 0$ See Fig. 10, left pane. For high λ_S, only one nonzero stable equilibrium is present, which corresponds to a situtation where parasites are outcompeted and go extinct. The point F_1 marks a fold bifurcation, after which, following the lower branch, a stable equilibrium emerges and parasites can coexist with replicators. Until the transcritical bifurcation (T), the system is bistable. The upper unstable branch departing from T corresponds to a situation where the system can be invaded by parasites (hence its instability), but is otherwise stable without (the other stable branch of the transcritical bifurcation is not included in the figure because equilibrium values for parasites become negative). For lower values of λ_S a Hopf bifurcation happens (H), with units of replication and parasites coexisting on a stable limit cycle, followed by a (possible) homoclinic bifurcation (h), when the minimum of the limit cycle hits the lower unstable manifold. After this value, depending on initial conditions (i.e. if parasites are present or not), the system will either converge to the state where no parasites are present, or will go extinct.

Lower a_{**}, different mutation rates and $\lambda_P \neq 0$ See Fig. 10, middle pane. Changing a_{**} and μ produce only quantitative differences, except at lower λ_S where the Hopf and the homoclinic bifurcation may disappear. However, setting $\lambda_P > 0$ changes the bifurcation plot qualitatively: both the transcritical and the fold bifurcation disappear, as well as the (unstable) equilibrium with no parasites at low λ_S, because parasites are always produced from units of replication. Despite this, parasites are present in very low numbers at higher λ_S, meaning that stallers effectively limit them.

High μ See Fig. 10, right pane. Pushing the system to higher μ has the effect of making $\bar{X}$ more dependent on λ_S as stallers are produced more often. F_2 marks the limit fraction of stallers that can be produced after which the system goes extinct (via fold bifurcation). For the highest μ parasites, even if arising as mutants of units of replication, cannot invade.

1.6 Snapshot of the field for a flat quasispecies

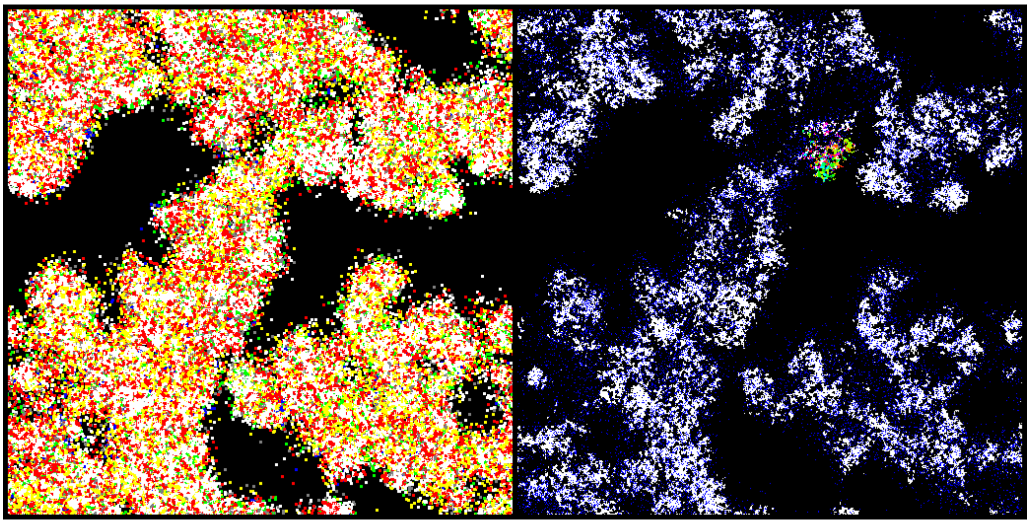

Figure 4: A snapshot of the field for the neutral quasispecies (7. in Table 1, in the main text): the figures correspond to the first two in Fig. 3. The colouring is the same. For the master sequence, the most abundant sequence in the field is used.

1.7 Sequences mentioned in Table 1

1. CCCCCCCCCCCCCGACACGGAAACGACGUGAGAGUCAUUAGAUAGGUGUC
2. CCCCCCCCCCCCCCGGCCGGAAACAACGUAAGAGCCAUUGUGUGGAUGCC
3. CCCCCCCCCCCAGCACCGGAAACAACGAAAGUACGCUGAAUGAGUGGUGC
4. ACCCCCCCCCCAGGCAACGAAAGACGAAAGAACGCCAUUGAGUGUGUGCC
5. CCCCCCCCCCGGCACAGGAAACAAAGCUAAAAUGCAGCCAUGCGGUGUGC
6. CCCCCCCCCCCGCAACGGCAACCGGAAAUCCAGCGACUUGAGUGUGUGCC
7. AACACUUCUACCCAGGCAAGGAAACACGGAAACAGCCAUCUUUUACUGCC
8. GAAAAAAAGAACAGAGCCAGGGGACACGUAAGGAGGCAUUAUACUAUGGC

References

Leigh Van Valen. A new evolutionary law. *Evolutionary Theory*, 1:30, 1973.
